# Impact of Structural Differences on the Modeled Cost-Effectiveness of Noninvasive Prenatal Testing

**DOI:** 10.1177/0272989X241263368

**Published:** 2024-08-02

**Authors:** Amber Salisbury, Alison Pearce, Kirsten Howard, Sarah Norris

**Affiliations:** Menzies Centre for Health Policy and Economics, Sydney School of Public Health, University of Sydney, Sydney, NSW, Australia; The Daffodil Centre, University of Sydney, a joint venture with Cancer Council NSW, Sydney, Australia; The Daffodil Centre, University of Sydney, a joint venture with Cancer Council NSW, Sydney, Australia; Sydney School of Public Health, University of Sydney, Sydney, NSW, Australia; Menzies Centre for Health Policy and Economics, Sydney School of Public Health, University of Sydney, Sydney, NSW, Australia; Menzies Centre for Health Policy and Economics, Sydney School of Public Health, University of Sydney, Sydney, NSW, Australia

**Keywords:** noninvasive prenatal testing, NIPT, prenatal screening, health economic modeling, economic evaluation, structural uncertainties, decision tree, microsimulation, cost-effectiveness analyses, cost-utility analyses

## Abstract

**Background:**

Noninvasive prenatal testing (NIPT) was developed to improve the accuracy of prenatal screening to detect chromosomal abnormalities. Published economic analyses have yielded different incremental cost-effective ratios (ICERs), leading to conclusions of NIPT being dominant, cost-effective, and cost-ineffective. These analyses have used different model structures, and the extent to which these structural variations have contributed to differences in ICERs is unclear.

**Aim:**

To assess the impact of different model structures on the cost-effectiveness of NIPT for the detection of trisomy 21 (T21; Down syndrome).

**Methods:**

A systematic review identified economic models comparing NIPT to conventional screening. The key variations in identified model structures were the number of health states and modeling approach. New models with different structures were developed in TreeAge and populated with consistent parameters to enable a comparison of the impact of selected structural variations on results.

**Results:**

The review identified 34 economic models. Based on these findings, demonstration models were developed: 1) a decision tree with 3 health states, 2) a decision tree with 5 health states, 3) a microsimulation with 3 health states, and 4) a microsimulation with 5 health states. The base-case ICER from each model was 1) USD$34,474 (2023)/quality-adjusted life-year (QALY), 2) USD$14,990 (2023)/QALY, (3) USD$54,983 (2023)/QALY, and (4) NIPT was dominated.

**Conclusion:**

Model-structuring choices can have a large impact on the ICER and conclusions regarding cost-effectiveness, which may inadvertently affect policy decisions to support or not support funding for NIPT. The use of reference models could improve international consistency in health policy decision making for prenatal screening.

**Highlights:**

- NIPT is a clinical area in which a variety of modeling approaches have been published, with wide variation in reported cost-effectiveness.
- This study shows that when broader contextual factors are held constant, varying the model structure yields results that range from NIPT being less effective and more expensive than conventional screening (i.e., NIPT was dominated) through to NIPT being more effective and more expensive than conventional screening with an ICER of USD$54,983 (2023)/QALY.
- Model-structuring choices may inadvertently affect policy decisions to support or not support funding of NIPT. Reference models could improve international consistency in health policy decision making for prenatal screening.

Health technology assessment (HTA) agencies provide advice on the funding of health technologies in many countries^1^ and typically rely on economic models to evaluate the comparative costs and benefits of these technologies. The stages of building a model are 1) selecting the analytical methods, 2) creating the structure, and 3) populating the model.^2^ As there is seldom complete information regarding the technology and how it will be used in clinical practice, different choices can be made at each stage of model development, which can generate significant uncertainty for decision makers.

The 3 main types of uncertainty in models are methodological, parameter, and structural.^3^ Methodological uncertainty includes differences in the analytic methods or the approach taken to build the model: the perspective chosen, the discount procedure/rate applied, or the way health gains are valued. Parameter uncertainty deals with the precision of the numerical value of inputs and is related to data quality. Structural uncertainty describes assumptions and simplifications within the model and the relationship between input parameters. The definition of structural uncertainty is often interpreted differently, resulting in apparent overlap between the different types of uncertainty.

In this article, we adopt an operational interpretation of structural uncertainty, which encompasses aspects of uncertainty that are often overlooked. Different choices can be made at each step of the structuring process, each leading to uncertainties. The first step is conceptual modeling, which involves selecting clinically/economically important health states and relevant patient attributes to include within the model. The second step is to make structural choices, which determine how model inputs are interrelated and estimated, such as the relationship between time and risk of an event, or the survival model used. The last step is the choice of modeling technique, for example, choosing between a decision tree or a Markov model. This choice reflects the complexity of the model and is influenced by decisions made in the previous steps. In considering the difference between structural and parameter uncertainty, we have taken the view that selection of patient attributes (including sociodemographic attributes such as age or gender, clinical attributes such as comorbidities or previous diseases, and lifestyle and behavioral attributes such as smoking or alcohol consumption) for inclusion within the model represents structural uncertainty, whereas the values assigned for each of these attributes represents parameter uncertainty. The approaches for handling parameter and methodological uncertainty are well established in the literature, while approaches for handling structural uncertainty are not.^4,5^

Differences in model structure are known to affect model predictions. In economic evaluations of lapatinib for breast cancer, different Markov model structures (alternative health states and transitions) resulted in a wide range of incremental cost-effective ratios (ICERs), differing by more than USD$250,000 (2016)/quality-adjusted life-year (QALY).^6^ Similarly, when evaluating adjuvant endocrine breast cancer treatment, changing the number of health states resulted in a 21.7% change in life-years gained and a 16.8% change in the ICER.^7^ A more recent study explored the impact of different modeling approaches on modeled results within diabetes and found microsimulation models generated a mean of 3.41 life-years more than cohort simulation models did.^8^ These studies demonstrate model structure has the potential to affect results and conclusions regarding cost-effectiveness, which may in turn affect funding decisions.

Noninvasive prenatal testing (NIPT) is a clinical area in which a variety of modeling approaches have been published, with wide variation in the modeled cost-effectiveness ratios. NIPT uses fetal DNA circulating in maternal plasma to detect 3 common trisomies (T21, Down syndrome; T18, Edwards syndrome; and T13, Patau syndrome) in the fetus.^9^ NIPT can be offered as a first-line test, replacing combined first-trimester screening (cFTS; ultrasound measurement of fetal nuchal translucency and maternal serum biochemical marker evaluation of β–human chorionic gonadotropin and pregnancy-associated plasma protein A) or as a second-line test after cFTS. It is claimed that NIPT offers a higher detection rate and lower false-positive rate compared with conventional screening methods.^10^ A previous systematic review identified 16 economic evaluations of NIPT with differing cost-effectiveness results.^11^ Fourteen studies assessed NIPT as a first-line test, and of these, 9 studies found NIPT was more expensive and more effective, 4 studies found NIPT was dominant (i.e., more effective and less expensive), and 1 found it was dominated (i.e., less effective and more expensive). Fifteen studies evaluated NIPT as a second-line test, and of these, 8 studies found NIPT was more expensive and more effective, 3 studies found NIPT was dominant, 1 study found NIPT was dominated, and 3 studies found NIPT was less effective and less expensive. The review identified variations in the time horizon, health outcomes, and contextual factors (such as year of analysis and currency) across the included studies, which the authors suggested were likely to have caused the different results. Other reviews of NIPT economic evaluations have assessed the cost-effectiveness of NIPT^11–13^ and the extent to which evaluations have accounted for the resourcing needed to provide counseling alongside testing.^14^ To our knowledge, no published economic evaluation or review has explored the impact of different structural choices on the modeled cost-effectiveness of NIPT and, by extension, the extent to which such choices represent a source of uncertainty that has not been accounted for by decision makers.

## Methods

### Research Aims

1. To describe differences in model structure for economic evaluations of NIPT for chromosomal abnormalities
2. To compare the impact of different model structures on modeled results

The analysis was performed in the following stages:

A. Systematic review of the literature to describe the structure of published economic models of NIPT
B. Development of new models evaluating NIPT for T21 with alternative structures, populated with parameters taken from the same sources (identified via supplementary targeted literature searches)
C. Comparison of modeled results from alternative model structures

### Stage A: Review of NIPT Model Structures

#### Information sources

A broad systematic review of economic evaluations of genetic and genomic tests was conducted as background research for a number of studies, including the current study. For the broader review, systematic searches were conducted in 4 databases: EMBASE, Medline, SCOPUS, and EconLit. The systematic search strategy for the published literature was based on 2 concepts: 1) genetic/genomic testing (including specific search terms for NIPT) and 2) economic evaluations. The search span was January 1, 2010, to June 15, 2022. Search terms are presented in the Supplementary Materials (Table S1). An additional search of HTA agency Web sites was performed in January 2023, using search terms for prenatal testing. Bibliographic review of included reviews and HTA reports was undertaken to capture additional studies that had not been identified in the electronic literature search.

#### Eligibility criteria

Model-based economic evaluations of NIPT that considered the costs and consequences of NIPT were eligible for inclusion, namely, cost-effectiveness (CEA), cost-utility (CUA), cost-benefit, cost-consequence, and threshold analyses. Trial-based studies, costing studies, budget impact analyses, nonhuman, and non-English studies were excluded.

#### Quality assessment

Quality assessment was performed using the Drummond checklist.^15^ A rating scale was used to attribute a possible score of 1 to each item on the checklist.^16^ The aggregate results provide an assessment of quality: poor (1–3 points), average (4–7 points), and good (8–10 points). The authors preagreed that studies scoring between 1 and 3 points would be excluded from the final analysis.

#### Data extraction

Study characteristics were extracted by a single reviewer (A.S.) and checked by a second reviewer (S.N.). The data extraction template was based on the Methods section of the CHEERS checklist^15^ and included the base-case results of each study. Study characteristics included country, study type, perspective, time horizon, clinical condition, discount rates, primary outcome measure(s), and type of sensitivity analysis. For each included study, a single reviewer (A.S.) documented elements of model structure within an Excel sheet and recorded whether study authors justified their chosen modeling approach, tested the impact of different modeling approaches within a sensitivity analysis, and/or provided a discussion of the potential impacts of their choices regarding model structure. For identified CUAs, the value of utility weights for a given health state, the source of each utility weight, and the method used to derive utility weights were also extracted.

#### Comparison of results from included studies

The base-case ICERs of included studies assessing NIPT as a second-line test against conventional screening were compared using a cost-effectiveness plane. We focused on second-line testing as a previous review^11^ identified a wider range of cost-effectiveness outcomes when considering NIPT as a second-line test as opposed to a first-line test. In addition, second-line NIPT has been considered for government funding in Australia^16^ and is therefore particularly relevant to our local policy context. It should be noted that “conventional screening” differed among the included studies, and where multiple comparator screening strategies were evaluated, the results for combined FTS against second-line NIPT were presented. To facilitate direct comparison of the published ICERs, reported incremental costs were converted to USD (2023) using Organisation for Economic Co-operation and Development (OECD) purchasing power parity^17^ and inflators from the total health price index.^18^

### Stage B: Building Alternative Model Structures

Our model-structuring process (model conceptualization, structural choices, and choice of modeling technique) was informed by the systematic review in stage A and consultation with experts including obstetricians (A.S., L.B.) and health economics/policy experts (K.H., A.P., S.N.). Additional advice on clinical practice was sought from 2 obstetricians, a genetic counselor, a midwife, and an individual with lived experience.

The expert consultation was crucial for determining a model structure that maximized the fit to pathology and practice by incorporating all relevant health states and patient attributes, given the available data. The literature review identified common NIPT model structures, enabling our selection of structures for comparison.

Four demonstration models with different structures were developed within TreeAge, comparing 3 versus 5 health states, and a decision tree approach versus microsimulation. Each of these was considered an appropriate model structure for our research purposes given the available evidence, expert opinion, and policy context (described in the “Results” section).

We defined the intervention as second-line NIPT. cFTS results were classified as high (risk score of >1:10), intermediate (risk score of >1:300), or low risk. It was assumed that women with a high risk result are offered invasive diagnostic testing, women with an intermediate risk result are offered NIPT, and women with a low risk result are not offered further testing. Women with a positive NIPT result are subsequently offered an invasive test. We have defined the comparator as conventional screening, in which women with a cFTS test risk of >1:300 are offered invasive diagnostic testing.

For each model structure, the population, comparator, time horizon, year of analysis, currency, and other core components were held constant to enable a comparison of the extent to which structural variations affected the modeled results. Nonetheless, the core components of each model were based on the clinical context in Australia and advice provided by 2 local obstetricians. The core components selected for the demonstration models, with justifications for these choices, are shown in Table 1.

**Table 1:** Core Components of Models Evaluating NIPT against cFTS

Component		Justification
Population	All singleton pregnancies regardless of trisomy risk	Simplification as different data are required for multiple pregnancies and these data are less reliable^19^
Setting	Australia	Australian context
Timing	All pregnant women enter at first trimester only	Simplification
Intervention	cFTS followed by NIPT in cFTS intermediate-risk pregnancies and invasive testing in cFTS high-risk pregnancies	Second-line testing is the most likely option to be funded in Australia
Comparator	cFTS with no NIPT and invasive testing in intermediate- or high-risk pregnancies	Current publicly funded testing in Australia
cFTS intermediate-risk cutoff	cFTS trisomy risk score of >1:300	Based on clinical advice and local hospital guidelines^20^
cFTS high-risk cutoff	cFTS trisomy risk score of >1:10	Based on clinical advice, this value ranges from 1/10 to 1/100; 1/10 is the conservative approach
Perspective	Health care funder	Local HTA guidelines^4^ as we are interested in how modeling informs decision making
Types of aneuploidies	T21	Simplification; T21 is being used as the case-study, but there will be implications for other uses of NIPT
Time horizon	Pregnancy duration	Simplification
Discount rate	None	Time horizon is less than 1 y
Currency	AUD and USD	AUD is presented as the model is set within the Australian context; costs are converted to USD to enable comparisons with other studies
Year of analysis	2023	Present context

AUD, Australian dollars; cFTS, combined first-trimester screening; HTA, health technology assessment; NIPT, noninvasive prenatal testing; USD, US dollars.

Each model structure was populated with consistent parameters for costs, clinical inputs, utility weights, and transition probabilities. Clinical parameters were obtained from a large, randomized control trial,^10^ and costs were obtained from the Australian Medical Benefits Schedule (see Supplementary Materials, Tables S5 and S6). A comprehensive supplementary literature review was conducted to identify appropriate utility weights. Based on this review, we used the utilities presented by Kuppermann et al.^21^ (Table 2). Details can be found within the Supplementary Material (section C).

**Table 2:** Utility Weights Used within Each Model

Health State	Utility Weight	Source
T21 birth	0.655	Kuppermann 2016^21^
Unaffected birth	1	Assumption
Termination	0.8	Kuppermann 2016^21^
Procedure-related loss	0.744	Kuppermann 2016^21^
Spontaneous loss	0.744	Kuppermann 2016^21^

All costs were converted to USD (2023) using the OECD purchasing power parity^21^ (1 AUD 2023 = 0.705 USD 2023).

Key assumptions made in each model are as follows:

- Patients receive an initial GP appointment to discuss genetic testing options.
- Patients with a positive result from screening have an appointment with their obstetrician, which includes genetic counseling.
- Patients who undergo invasive testing are provided with genetic counseling after testing, from the obstetrician.
- Patients with inconclusive NIPT results go directly to invasive testing.
- Invasive testing is 100% sensitive and specific.

Internal validation involved ensuring models maintained logical coherence when adjusting costs and utilities while also verifying that 1-way sensitivity analyses and scenario analyses yielded expected outcomes. External validation involved comparing the results of our models to the Australian models identified within the main review.

### Stage C: Comparison of ICERs from Different Model Structures

For each model, we present the following results:

- the incremental cost/T21 detected,
- the incremental cost/procedure-related loss (PRL) avoided, and
- the incremental cost/QALY.

The results of each model are compared by examining the overall outcome of the model (i.e., NIPT dominant, NIPT dominated, NIPT more expensive and more effective, NIPT less expensive and less effective). One-way sensitivity analyses were performed on the following parameters using plausible extreme values: uptake rates of cFTS, NIPT, and invasive testing (after an intermediate-risk cFTS result, after a high-risk cFTS result, and after a positive-risk NIPT result) and cFTS and NIPT sensitivity and specificity. Distributions for the following parameters were included in the probabilistic analysis: spontaneous loss, NIPT and cFTS sensitivity and specificity, rates of pregnancy termination, and uptake rates of cFTS, NIPT, and invasive testing (after an intermediate-risk cFTS result, after a high-risk cFTS result, and after a positive-risk NIPT result).

The funding source had no role in the study.

## Results

### Stage A: Review of NIPT Model Structures

**Figure 1:**
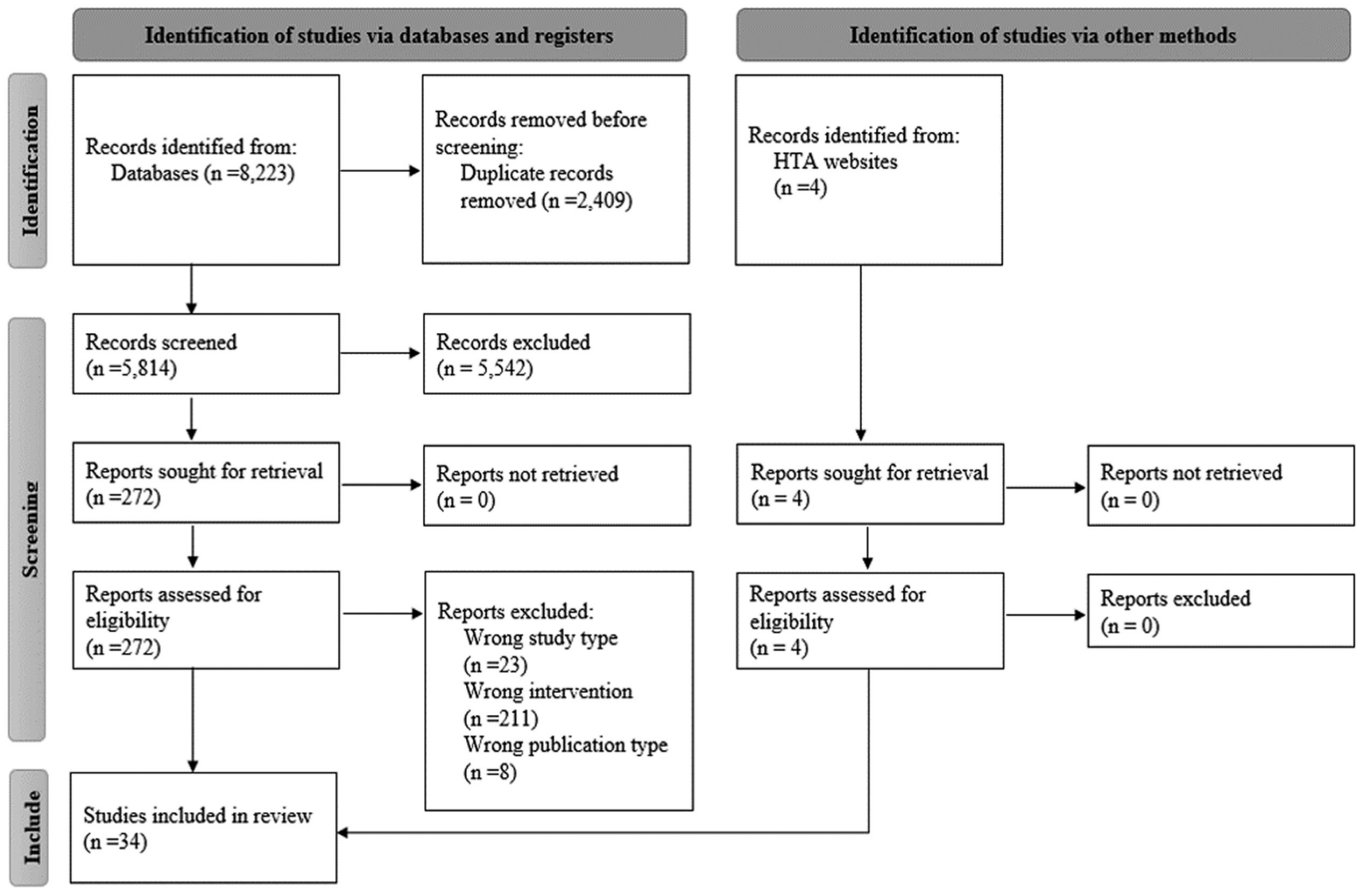
PRISMA flow diagram.

The database search yielded 8,223 records published between January 1, 2010, and June 15, 2022 (Figure 1). After removing duplicates, 5,814 records were included in the title/abstract screen, and 272 articles were obtained for full-text review. Based on full-text review, 30 studies met the inclusion criteria for this study, and 4 additional HTAs of NIPT were identified through HTA Web sites. Ten studies were scored as average quality, and 24 were scored as good quality. No studies were scored as poor quality, and thus all studies were included in this analysis. The quality assessment can be found in the Supplementary Materials (section D).

#### Study characteristics

Thirty-four studies were included, with studies conducted in the United States (*n* = 10, 29%), Canada (*n* = 7, 21%), Belgium (*n* = 4, 12%), China (*n* = 4, 12%), and other countries (*n* = 9, 26%). Most studies conducted a CEA from a health care funder perspective, over the duration of pregnancy, and focused on T21 (Table 3). More than 60% of studies included uptake rates; however, these rates were mostly based on conventional screening evidence. See Supplementary Materials (Table S10) for more details.

**Table 3:** Basic Study Characteristics of Economic Models Comparing NIPT to Conventional Screening

	Response	
Study Characteristic (*N* = 34)	*n*	%
Study type		
CEA	23	68
Cost and consequence	4	12
CUA	3	9
Threshold analysis	3	9
CBA	1	3
Perspective		
Health care funder	20	59
Health care funder + patient	8	24
Societal	4	12
Societal and health care funder	2	6
Time horizon		
Pregnancy until birth	26	76
5 y	1	3
18 y	1	3
Lifetime	6	18
Condition		
T21	23	68
T21, T18, and T13	8	24
T21, T18, T13, and monosomy X	2	6
T21, T18, T13, sex chromosomes, and microdeletions	1	3
Uptake included?		
Yes, with evidence	23	68
Yes, without evidence	6	18
No	5	15

CBA, cost-benefit analysis; CEA, cost-effectiveness analysis; CUA, cost-utility analysis; NIPT, noninvasive prenatal testing; T13, trisomy 13; T18, trisomy 18; T21, trisomy 21.

#### Structural elements

The structural elements identified from the included studies are shown in Table 4. The number of health states included within the models ranged from 3 to 7, with 5 being the most common (53% of models). The 3 most common health states were live birth (unaffected), live birth (T21), and procedure-related loss (PRL), followed by “spontaneous loss” and “termination.” The modeling approaches included decision trees (*n* = 22, 65%), Markov models (*n* = 7, 21%), and microsimulations (*n* = 5, 15%). Four studies justified their modeling approach, and 2 studies included a discussion of their modeling approach. No study tested the impact of their approach on their modeled results.

**Table 4:** Structural Elements of Identified Economic Models^a^

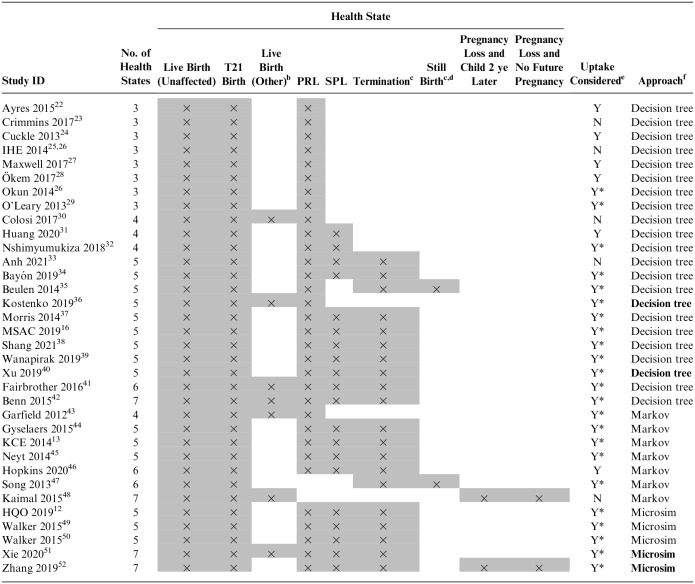

Microsim; microsimulation, N; no, PRL; procedure-related loss, SPL; spontaneous pregnancy loss, Y; yes.

a Studies have been ordered by number of health states and then by modeling approach (decision tree, Markov model, microsimulation). This ordering attempts to reflect increasing model complexity. Gray highlighting with a cross indicates the health state has been included within the identified model.

b “Other” refers to the following conditions: microdeletions, sex chromosomes, and/or variants of unknown significance.

c Health states within this category are either labeled affected and/or not affected or the health state is unspecified. This column combines 3 different health states.

d Both spontaneous loss and stillbirth describe pregnancy loss, but they differ according to when the loss occurs (before and after 20 wk).

e “Y*” indicates uptake has been considered with evidence, “Y” indicates uptake has been considered without evidence, and “N” means uptake has not been considered.

f Studies that provided justification for their modeling approach and/or included potential impacts of the structure within the discussion are marked in bold (e.g., **Microsim** or **Decision tree)**.

#### Results of included studies

**Figure 2:**
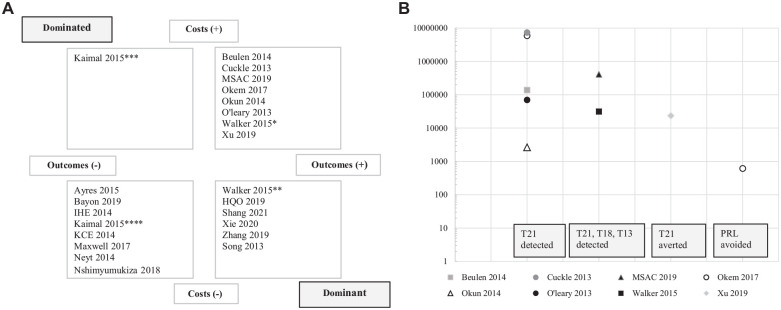
(a) Cost-effectiveness plane for studies evaluating second-line NIPT against conventional screening. Incremental cost-effectiveness ratios for 4 different outcomes are presented: T21 detected; T21, T18, and T13 detected; T21 averted and PRL avoided. (b) Detailed view of the upper right-hand quadrant of the cost-effectiveness plane in Figure 2a. Each outcome is presented on a separate horizontal axis, and results are displayed using a log scale in 2023 USD. NIPT, noninvasive prenatal testing; PRL, procedure-related loss; T, trisomy. *Payer perspective. **Government and societal perspective. ***Twenty- and 30-year-olds. ****Forty-year-olds.

A comparison of the results of second-line NIPT against conventional screening is presented in Figure 2 (*n* = 23). Six studies found NIPT was dominated (lower right-hand quadrant), 1 study found NIPT was dominant (upper left-hand quadrant), 8 studies found NIPT was less expensive and less effective (lower left-hand quadrant), and 8 studies found NIPT was more expensive and more effective (upper right-hand quadrant) (Figure 2a). There were large variations in the ICERs within the upper right-hand quadrant (Figure 2b).

#### Cost-utility analyses

Three CUAs were identified.^33,48,52^ All studies used utility weights from the perspective of the mother and were derived from time-tradeoff studies.^53^ The utilities for each health state and sources can be found within the Supplementary Materials (Table S11).

### Stage B: Building Alternative Model Structures

#### Model conceptualization

Based on the research question and expert consultation, the following patient attributes were considered relevant for inclusion:

- Impact of maternal age on the risk of Down syndrome
- Impact of maternal age on the risk of spontaneous loss

The following health states/events were considered relevant for inclusion:

- Final health states: live birth (T21), live birth (unaffected), termination, spontaneous loss, procedure-related loss
- Intermediate events: the anxiety and wait times associated with receiving a false-positive or inconclusive result

The systematic review found a large proportion of published models did not include termination and/or spontaneous loss. We built models to compare the impact of including all health states considered to be clinically relevant and a simplified model that does not include termination or spontaneous loss. Due to our decision to hold the time horizon constant across the models being explored, health states beyond 1 y (e.g., giving birth to a child 2 y later or no future pregnancy) were not included in our models.

A large number of the included published models did not include risks based on maternal age. We built models to compare the impact of including or excluding these risks. Due to a lack of available data, intermediate outcomes were not included within the published models or within our models.

#### Structural choices

Due to the short-term nature of our model (as decided in the methodological stage), extrapolation was not used, nor was survival analysis. There are no time dependencies within the model.

#### Modeling techniques

The choice of modeling technique stems from the model conceptualization. The least complicated but most feasible technique should be chosen.^54^ Microsimulations are best used for complex models with large variability in the cohorts.^55^ Consequently, we used a microsimulation approach to build the models that incorporate risks based on maternal age. When these risks were not incorporated, we used a decision tree, as there was no longer a need to use a microsimulation. An alternative approach to capturing different risks based on maternal age could be through subgroup analysis or by creating subtrees for different ages within a decision tree.

Within the systematic review, decision trees, Markov models, and microsimulations were identified. Markov models are best used for long-term chronic diseases, which incorporate time dependencies.^55^ The identified Markov models did not incorporate any time dependencies and were not fundamentally different from the identified decisions trees. As we were not incorporating any time dependencies, a Markov model was not built.

#### Overview of demonstration models

Four alternative structures were created (Table 5), which differed by number of health states (3 or 5) and modeling approach (decision tree or microsimulation, reflecting the difference in incorporating risks based on maternal age). Diagrams of the model structures can be found in the Supplementary Materials (section H).

**Table 5:** Alternative Model Structures to Compare Second-Line NIPT against cFTS

Model ID	Modeling Approach	No. of Health States	Included Health States
Model D3	Decision tree	3	Live birth (unaffected), live birth (T21), PRL
Model D5	Decision tree	5	Live birth (unaffected), live birth (T21), PRL, spontaneous loss, termination
Model M3	Microsimulation	3	Live birth (unaffected), live birth (T21), PRL
Model M5	Microsimulation	5	Live birth (unaffected), live birth (T21), PRL, spontaneous loss, termination

cFTS, combined first-trimester screening; NIPT, noninvasive prenatal testing; PRL, procedure-related loss.

### Stage C: Comparison of Results

The ICERs varied among the 4 models for each of the 3 outcomes evaluated (T21 detected, PRL avoided, and QALY). These results are presented in Table 6.

**Table 6:** Results from Different Model Structures Evaluating cFTS versus NIPT from Pregnancy until Birth

Model ID	Screening Strategy	Cost, USD 2023 (AUD 2023)	Incremental Cost, USD 2023 (AUD 2023)	Effect	Incremental Effect	ICER, USD 2023 (AUD 2023)	Outcome
Effect: T21 detected							
Model D3	cFTS	100.62 (142.81)		0.001578		66,936 (93,141)	NIPT is more expensive and more effective
	NIPT	101.43 (143.95)	0.80 (1.14)	0.001590	0.000012		
Model D5	cFTS	173.98 (246.92)		0.001578		69,872 (96,873)	NIPT is more expensive and more effective
	NIPT	174.82 (248.11)	0.84 (1.19)	0.001590	0.000012		
Model M3	cFTS	126.02 (178.85)		0.001613		−95,120 (−139,000)	NIPT is dominated (more expensive and less effective)
	NIPT	126.97 (180.20)	0.95 (1.39)	0.001603	−0.000010		
Model M5	cFTS	198.79 (282.13)		0.001613		−156,700 (−110,621)	NIPT is dominated (more expensive and less effective)
	NIPT	199.89 (283.70)	1.11 (1.57)	0.001603	−0.000010		
Effect: PRL avoided							
Model D3	cFTS	100.62 (142.81)				8,827 (12,528)	NIPT is more expensive and more effective
	NIPT	101.43 (143.95)	0.80 (1.14)	0.000013	0.000091		
Model D5	cFTS	173.98 (246.92)		0.000104		9,214 (13,030)	NIPT is more expensive and more effective
	NIPT	174.82 (248.11)	0.84 (1.19)	0.000013	0.000091		
Model M3	cFTS	126.02 (178.85)		0.0000800		12,353 (18,177)	NIPT is more expensive and more effective
	NIPT	126.96 (180.20)	0.95 (1.39)	0.0000033	0.000077		
Model M5	cFTS	198.79 (282.13)		0.0000800		14,366 (20,439)	NIPT is more expensive and more effective
	NIPT	199.89 (283.70)	1.11 (1.57)	0.0000033	0.000077		
Effect: QALY							
Model D3	cFTS	100.62 (142.81)		0.9989434		34,474 (48,844)	NIPT is more expensive and more effective
	NIPT	101.43 (143.95)	0.80 (1.14)	0.9989667	0.0000233		
Model D5	cFTS	173.98 (246.92)		0.9915223		14,990 (21,236)	NIPT is more expensive and more effective
	NIPT	174.82 (248.11)	0.84 (1.19)	0.9915578	0.0000560		
Model M3	cFTS	126.02 (178.85)		0.9989341		54,983 (80,431)	NIPT is more expensive and more effective
	NIPT	126.96 (180.20)	0.95 (1.39)	0.9989515	0.0000173		
Model M5	cFTS	198.79 (282.13)		0.9916393		−16,032 (−22,676)	NIPT is dominated (more expensive and less effective)
	NIPT	199.89 (283.70)	1.11 (1.57)	0.9915702	−0.000069		

AUD, Australian dollars; cFTS, combined first-trimester screening; ICER, incremental cost-effectiveness ratio; NIPT, noninvasive prenatal testing, PRL, procedure-related loss; QALY, quality-adjusted life-year; T21, trisomy 21; USD, US dollars. ICER refers to the cost/effect. Model 1: 3 health states and a decision tree; model 2: 5 health states and a decision tree; model 3: 3 health states and a microsimulation; model 4: 5 health states and a microsimulation.

#### Sensitivity analysis

One-way and probabilistic sensitivity analyses are shown in the Supplementary Materials (Tables S12, S13, and S14). Uptake of NIPT and invasive testing were found to have the greatest impact on results. Uptake of cFTS was found to have minimal impact on results.

## Discussion

This study identified a large number of economic evaluations of NIPT with different model structures and cost-effectiveness results. By developing new models that hold model inputs constant but vary key structural components of number of health states (3 v. 5) and modeling approach (decision tree v. microsimulation), we demonstrate that variations in model structure alone can have large effects on cost-effectiveness results. For example, with a CUA, the ICERs ranged from NIPT being dominated (model M5), to being cost-effective (at the commonly referenced threshold of USD$50,000/QALY,^56^ model D3 and D5) to being not cost-effective at more than USD$50,000/QALY (model M3). These findings demonstrate that model structure in this context represents a significant, previously unrecognized, source of uncertainty for decision makers who rely on model findings.

The systematic review identified 34 economic models comparing NIPT against conventional screening methods. Twelve additional studies were identified compared with recently conducted reviews,^11,12^ highlighting the large volume of research in this area. Most studies conducted CEAs from the health care funder perspective and used a short time horizon. Although guidelines recommend conducting a CUA where possible,^4^ most studies justified a CEA due to the paucity of published studies assessing utilities, particularly in the context of intermediate outcomes (such as the psychological impact of a false positive). In addition, QALYs have limitations in adequately capturing the benefits associated with new life,^57^ and ethical concerns arise when valuing the life of a child with Down syndrome.^58^

Based on the included CUAs,^7,9,10^ the main source of utility weights was a US time-tradeoff study. Although that study attempted to capture the impact of intermediate outcomes, only minor decrements in utility were found, and these findings do not align with the broader qualitative literature.^12,16^ The authors of the time-tradeoff study hypothesize that the lack of impact is likely due to the methodology used, with participants focusing on final outcomes and overdiscounting intermediate outcomes. To adequately capture intermediate outcomes, it may be necessary to consider alternative valuation methods, such as discrete choice experiments.^14^

Our systematic review expands on previous reviews by extracting additional information on health states. A range of health states were identified, with the most common health states being live birth (T21), live birth (unaffected), and procedure-related loss, followed by termination and spontaneous loss health states, although they were not included in more than 40% of identified models. This study created demonstration models to compare the impact of including 3 versus 5 health states (i.e., adding termination and spontaneous loss to the model). When using a decision tree and 3 health states, the ICER was approximately USD$35,000 (2023)/QALY, indicating that decision makers may consider funding NIPT. Under a decision tree with 5 health states, NIPT became considerably more cost-effective and thus more likely to be funded, with the ICER decreasing to USD$15,000 (2023)/QALY. Adding 2 health states resulted in small absolute variations in QALYs; however, a small incremental QALY gain led to a relatively large impact on the ICER. The QALY gain was likely driven by the addition of termination as a health state, which reflects the decision made by most women upon receiving a T21 diagnosis.^41,42^ Cost-utility analyses of NIPT are sensitive to the number of health states included in the model, and restricting the model to 3 health states is likely to be an oversimplification.

The demonstration model was less sensitive to the number of health states within a CEA. When termination and spontaneous loss were added to the model, there was minimal change in the ICER, most likely due to less differentiation between health states. Within the CEA, spontaneous loss, procedure-related loss, and termination were associated with the same cost, while within the CUA, the same cost was applied to these states, but different utility weights were assigned. An additional finding from this study was that the results and conclusions of the economic models of NIPT were highly dependent on the outcomes being analyzed. When considering cost/T21 detected, 50% of model structures resulted in NIPT being dominated. When considering cost/PRL avoided, none of the model structures found NIPT to be dominated. When considering cost/QALY, 25% of model structures found NIPT to be dominated. This study supports the conclusions made by previous reviews,^11,12^ highlighting the challenges arising from the choice of outcome.

A range of modeling techniques (decision tree, Markov model, microsimulation) were identified across included studies. Only a small proportion of studies justified their approach or included a discussion of the possible impacts of their approach on the modeled results. No studies assessed the impacts of the chosen approach within a sensitivity analysis. The current study assessed the impact of using either a decision tree or a microsimulation. The fundamental difference between the 2 approaches was that the decision tree did not consider population heterogeneity, whereas the microsimulation allowed for the incorporation of risks based on maternal age. When looking at cost/T21 detected and using a microsimulation, NIPT was more expensive and less effective and unlikely to be funded. In contrast, a decision tree approach resulted in ICERs below USD$70,000 (2023)/T21 detected, making NIPT more likely to be funded. Microsimulations are recommended for complex interventions because they can incorporate patient heterogeneity and time dependencies.^15^ In the context of prenatal screening, patient heterogeneity plays a large role in clinical effectiveness, as the risk of having a child with Down syndrome varies with maternal age.^59^ Given the clinical significance of these risks and proven impact on modeled results, we recommend including them within the model.

A previous review of NIPT economic models concluded variations in findings across studies can be primarily attributed to methodological and contextual factors.^11^ The current study demonstrates that when employing the same methodology and contextual framework, variations in model structure are associated with large differences in ICER results. However, there is a lack of consensus on how to handle structural uncertainty.^4,5,60^ Based on the current work, we created a summary comparison of alternative approaches to managing structural uncertainty, including scenario analysis, model averaging, and value-of-information analysis (Table 7). There is no universal solution, as each available method can have different potential benefits. The most comprehensive option appears to be disease-specific reference models, which have been suggested for complex disease areas.^66,67^ They are multiuse models that aim to streamline the model development process and improve model credibility.^63^ However, the development of reference models requires a large upfront investment. Prenatal screening presents a complex decision-making area, involving multiple conditions and outcomes. This complexity suggests reference models could be valuable and worth the investment. Further research is needed to assess the feasibility of reference models in the context of prenatal screening and genetic/genomic testing more broadly. The incorporation of reference models into current HTA processes warrants further exploration.

**Table 7:** Overview of Methods for Handling Structural Uncertainty and Their Potential Benefits as Judged by the Research Team

Method	Judgment of Workload to Build the Initial Model	Uncertainty Is Characterized	Single CE Estimate	Practical Guidance on Appropriate Model	Increase Model Transparency	Possible Efficiencies for Future Model Building	Case Studies
Scenario analysis	High	Yes			Yes		Le 2016,^6^ Frederik 2014,^7^ Petersohn 2021^64^
*Report alternative models based on different structural assumptions*							
Model averaging	High	Yes	Yes		Yes		Petersohn 2021^64^
*Weighting outcomes from a set of plausible models based on fit to observed data or expert opinion*							
Model parameterization	Moderate to high	Yes	Yes		Yes		Petersohn 2021^64^
*Adding parameters to the model that define alternative structural choices*							
Model registries	Low				Yes	Yes	Pillsbury 2014^65^
*Store models within an online open access model repository*							
Value-of-information analysis	Moderate to high	Yes			Yes		Strong 2014,^66^ Petersohn 2021^64^
*Quantifying the value of acquiring additional information*							
Model discrepancy analysis	Moderate to high	Yes			Yes		Strong 2012,^67^ James 2021^68^
*Quantifying the difference between the model evaluated at its “true” input values and the true value of the output quantity*							
Disease-specific reference models	Very high		Yes	Yes	Yes	Yes	Drummond 2003,^69^ Hiligsmann 2019^70^
*Multiuse models for use within a disease context*							

CE, cost-effectiveness.

### Limitations

The systematic search was limited to databases and HTA Web sites, potentially missing studies from other sources. Given the large number of studies included within the background systematic review, the quality assessment was limited to the Drummond checklist. We consider that this checklist provides an acceptable balance of quality assessment indicators versus efficiency. We aimed to assess the impact of structural uncertainty, rather than the cost-effectiveness of NIPT, and as a result a number of simplifications were made: we considered NIPT as a second-line test, over a short time horizon, limited the conditions assessed to T21, and we excluded the impact of intermediate outcomes. Although the models do not include T18 and T13, the results have implications for both conditions. It is likely that when considering additional complexities, structural variations would have a greater impact on results. For example, the impact of uncertain findings will be greater when considering expanded NIPT, and their inclusion is likely to affect CEA results. Similarly, including the possibility of another pregnancy in a longer-term model is likely to create further differences in modeled results. As with all models, we are restricted by the data available, and no model is a perfect reflection of reality. This article demonstrates how structure can affect results but does not demonstrate the impact of all model structures. Instead, it draws on the literature to select the most appropriate structures for comparison.

## Conclusion

This study summarizes the current evidence base for economic evaluations of NIPT and explores the impact of structural uncertainty on modeled results. Most studies conducted cost-effectiveness analyses, and only a small number conducted cost-utility analyses. There is a gap in the literature regarding the valuation of utilities for health outcomes in this context, particularly regarding intermediate outcomes. Published model structures differ in their level of complexity, with key variations in the number of health states and modeling approach. This study demonstrates that variations in model structure are associated with large differences in the ICER and resulting conclusions regarding cost-effectiveness. Policy makers should be aware that structural choices made by modelers may inadvertently affect decisions to support or not support funding for NIPT. The use of context-specific reference models could be explored to provide practical guidance on model selection.

## Supplemental Material

sj-docx-1-mdm-10.1177_0272989X241263368 – Supplemental material for Impact of Structural Differences on the Modeled Cost-Effectiveness of Noninvasive Prenatal Testing Supplemental material, sj-docx-1-mdm-10.1177_0272989X241263368 for Impact of Structural Differences on the Modeled Cost-Effectiveness of Noninvasive Prenatal Testing by Amber Salisbury, Alison Pearce, Kirsten Howard and Sarah Norris in Medical Decision Making
