# Supplementary material for: Impact of Structural Differences on the Modeled Cost-Effectiveness of Noninvasive Prenatal Testing
Source: Med Decis Making. 2024 Aug 2;44(7):811–27. doi: 10.1177/0272989X241263368 (PMC11492563; doi:10.1177/0272989X241263368)
Supplement: sj-docx-1-mdm-10.1177_0272989X241263368 – Supplemental material for Impact of Structural Differences on the Modeled Cost-Effectiveness of Noninvasive Prenatal Testing [file sj-docx-1-mdm-10.1177_0272989X241263368.docx]

# Supplementary materials

1. Search Terms

**Table S1 EMBASE search terms**

| **No.** | **Query** |
| --- | --- |
| 1 | high throughput sequencing'/exp OR 'high throughput sequenc*':ti,ab,kw OR 'next generation sequenc*':ti,ab,kw,de OR 'next-generation sequenc*':ti,ab,kw,de OR ngs:ti,ab,kw,de OR 'non-invasive prenatal test*':ti,kw,ab,de OR 'non invasive prenatal test*':ti,kw,de,ab OR nipt:ti,kw,de,ab OR 'noninvasive prenatal test*':ti,ab,de,kw OR metagenomic$:ti,ab,kw,de OR wgs:ti,ab,kw,de OR wes:ti,ab,kw,de OR germline:ti,ab,kw,de OR inherited:ti,ab,kw,de OR inheritable:ti,ab,kw,de OR 'genomic test*':ti,ab,kw,de OR 'gene* test*':ti,ab,kw,de |
| 2 | (('massive* parallel' OR panel OR deep OR 'whole genome' OR exome OR multigene OR 'multi gene' OR monogenic) NEAR/5 (sequenc* OR test*)):ti,ab,kw,de |
| 3 | #1 OR #2 |
| 4 | economic*:ti OR cost*:ti OR utilit*:ti OR 'quality adjusted life year:ti' OR qaly:ti OR 'life year saved':ti OR icer:ti OR value:ti OR hta:ti OR 'health technology assessment':ti OR markov:ti OR 'decision tree':ti OR 'outcome assessment':ti OR 'decision analy*':ti OR 'decision-analy*':ti OR 'cea':ti |
| 5 | #3 AND #4 |
| 6 | #5 NOT (('animal'/exp OR 'nonhuman'/exp OR 'rodent'/exp OR 'animal experiment'/exp OR 'experimental animal'/exp OR rat:ti,ab OR rats:ti,ab OR mouse:ti,ab OR mice:ti,ab OR dog$:ti,ab OR pig$:ti,ab OR porcine:ti,ab OR swine:ti,ab OR chick$:ti,ab) NOT 'human'/exp) AND [2010-2022]/py |
| 7 | #6 NOT ([conference abstract]/lim OR [conference review]/lim OR [editorial]/lim OR [erratum]/lim OR [letter]/lim OR [note]/lim OR [book]/lim OR 'case report'/de) AND [english]/lim |

**Table S2 MEDLINE search terms**

| **No.** | **Query** | |
| --- | --- | --- |
| 1 | High-Throughput Nucleotide Sequencing/ or high throughput sequenc*.mp. or next generation sequenc*.mp. or next-generation sequenc*.mp. or ngs.mp. or non-invasive prenatal test*.mp. or non invasive prenatal test*.mp. or nipt.mp. or noninvasive prenatal test*.mp. or metagenomic$.mp. or wgs.mp. or wes.mp. or germline.mp. or inherited.mp. or inheritable.mp. or genomic test*.mp. or gene* test*.mp. |  |
| 2 | ((massively parallel or panel or whole genome or exome or multigene or multi gene or monogenic) and (sequenc* or test*)).mp. |  |
| 3 | 1 or 2 |  |
| 4 | economic*.ti. or cost*.ti. or utilit*.ti. or quality adjusted life year.ti. or qaly.ti. or life year saved.ti. or icer.ti. or value.ti. or hta.ti. or health technology assessment.ti. or markov.ti. or decision tree.ti. or outcome assessment.ti. or decision analy*.ti. or decision-analy*.ti. or cea.ti. |  |
| 5 | 3 and 4 |  |
| 6 | limit 5 to (humans and yr="2010 - 2022") |  |

**Table S3 SCOPUS search terms**

| **No.** | **Query** |
| --- | --- |
| 1 | TITLE( "high throughput sequenc*" OR "next generation sequenc*" OR ngs OR "non invasive prenatal test*" OR nipt OR "noninvasive prenatal test*" OR metagenomic$ OR wgs OR wes ) OR (TITLE((germline OR inherited OR inheritable OR gene* OR genomic OR "massively parallel" OR panel OR "whole genome" OR exome OR multigene OR "multi gene" OR monogenic) AND (sequenc* OR test*))) AND ( TITLE ( economic* OR cost* OR utilit* ) ) AND ( LIMIT-TO ( PUBYEAR,2022) OR ( LIMIT-TO ( PUBYEAR,2021) OR LIMIT-TO ( PUBYEAR,2020) OR LIMIT-TO ( PUBYEAR,2019) OR LIMIT-TO ( PUBYEAR,2018) OR LIMIT-TO ( PUBYEAR,2017) OR LIMIT-TO ( PUBYEAR,2016) OR LIMIT-TO ( PUBYEAR,2015) OR LIMIT-TO ( PUBYEAR,2014) OR LIMIT-TO ( PUBYEAR,2013) OR LIMIT-TO ( PUBYEAR,2012) OR LIMIT-TO ( PUBYEAR,2011) OR LIMIT-TO ( PUBYEAR,2010) ) AND ( LIMIT-TO ( LANGUAGE,"English" ) ) |

**Table S4 ECON LIT search terms**

| **No.** | **Query** |  |
| --- | --- | --- |
| 1 | TI(Sequencing) OR TI(sequence analysis) OR TI(genomics) OR TI(genomic testing) OR TI(gene testing) OR AB(Sequencing) OR AB(sequence analysis) OR AB(genomics) OR AB(genomic testing) OR AB(gene testing)  Limits: 2010-2022 | |

1. Clinical parameters and costs

**Table S5 Clinical parameters for models comparing NIPT against current screening**

| **Parameter** | **Value 1** | **Source** |
| --- | --- | --- |
| Number of singleton pregnancies | 300,000 | Approximate number of births in Australia (AIHW 2022) |
| T21 viable foetus rate at first trimester | 0.299% | Maxwell 2016 |
| Spontaneous loss between 12 weeks and term (unaffected) | 3% | Avlos 2012 |
| T21 spontaneous pregnancy loss rate between 12 weeks and term | 43% | Morris 1999 |
| T21 termination rate | 93% | Maxwell 2015 |
| % chronic villus sampling | 24% | Norton 2011 |
| % amniocentesis | 76% | Norton 2015 |
| Chronic villus sampling PRL | 0.20% | Salomon 2019 |
| Amniocentesis PRL | 0.30% | Solomon 2019 |
| Overall PRL | 0.276% | Calculation |
| **cFTS** |  |  |
| Specificity | 94.6% | Norton 2015 |
| Sensitivity | 78.90% | Norton 2015 |
| Percentage who test high risk | 0.40% | Maxwell 2017 |
| PPV of those who test high risk | 45% | Maxwell 2016 |
| **NIPT T21** |  |  |
| Specificity | 99.9% | Norton 2015 |
| Sensitivity | 100% | Norton 2015 |
| Failure rate | 3% | Norton 2015 |
| **Uptake of screening tests** |  |  |
| Uptake of cFTS | 83.60% | Lindquist 2019 |
| Uptake of NIPT after cFTS | 100.00% | Assumption |
| ***Uptake of invasive testing*** |  |  |
| After unreportable NIPT | 100% | Assumption |
| After positive cFTS | 80% | Maxwell 2016 |
| After positive NIPT | 80% | Assumption |
| After high-risk cFTS | 90% | Maxwell 2016 |

Abbreviations: cFTS, combined first trimester screening, NIPT, non-invasive prenatal testing; PRL, procedure-related loss.

**Table S6a Costs for models comparing NIPT against current screening (AUD)**

| **Service** | **Fee** | **75% benefit** | **85% benefit** | **Chosen value** | **Source** |
| --- | --- | --- | --- | --- | --- |
| cFTS biochemical testing | $39.75 | $29.85 | $33.80 | $33.80 | MBS item 66750 |
| cFTS NT ultrasound | $72.85 | $54.65 | $61.95 | $61.95 | MBS item 55707 |
| Total cFTS |  |  |  | $95.75 |  |
| NIPT | $400 |  |  | $400 | MSAC Public summary document^17^ - Proposed MBS item descriptor |
| Amniocentesis | $67.10 | $50.35 | $57.05 | $57.05 | MBS item 16600 |
| Chronic villus sampling | $128.85 | $96.65 | $109.55 | $109.55 | MBS item 16603 |
| Associated ultrasound | $113.55 | $85.20 | $96.55 | $96.55 | MBS item 55054 |
| Pathology cytogenetics | $394.55 | $295.95 | $335.40 | $335.40 | MBS item 73287 |
| Patient Episode Initiation | $2.40 | $1.80 | $2.05 | $2.05 | MBS item 73939 |
| Weighted invasive testing |  |  |  | $517.30 |  |
| GP consult | $39.75 |  |  | $39.75 | MBS item 23 |
| Obstetrician and genetic counselling initial | $91.80 | $68.85 | $78.05 | $78.05 | MBS item 104 |
| Obstetrician and genetic counselling subsequent | $46.15 | $34.65 | $39.25 | $39.25 | MBS item 105 |
| Hospital - Abortion W Gis | $3,250.96 |  |  | $3,250.96 | DRG O05Z Abortion W Gis |

Abbreviations: cFTS, combined first trimester screening; GP, general practitioner; NIPT, non-invasive prenatal testing; PRL, procedure-related loss.

**Table S7b Costs for models comparing NIPT against current screening (converted to USD)**

| **Service** | **Fee** | **75% benefit** | **85% benefit** | **Chosen value** | **Source** |
| --- | --- | --- | --- | --- | --- |
| cFTS biochemical testing | $28.01 | $21.03 | $23.82 | $23.82 | MBS item 66750 |
| cFTS NT ultrasound | $51.33 | $38.51 | $43.65 | $43.65 | MBS item 55707 |
| Total cFTS |  |  |  | $67.46 |  |
| NIPT | $281.84 |  |  | $281.84 | MSAC PSD - Proposed MBS item descriptor |
| Amniocentesis | $47.28 | $35.48 | $40.20 | $40.20 | MBS item 16600 |
| Chronic villus sampling | $90.79 | $68.10 | $77.19 | $77.19 | MBS item 16603 |
| Associated ultrasound | $80.01 | $60.03 | $68.03 | $68.03 | MBS item 55054 |
| Pathology cytogenetics | $278.00 | $208.52 | $236.32 | $236.32 | MBS item 73287 |
| Patient Episode Initiation | $1.69 | $1.27 | $1.44 | $1.44 | MBS item 73939 |
| Weighted invasive testing |  |  |  | $364.49 |  |
| GP consult | $28.01 |  |  | $28.01 | MBS item 23 |
| Obstetrician and genetic counselling initial | $64.68 | $48.51 | $54.99 | $54.99 | MBS item 104 |
| Obstetrician and genetic counselling subsequent | $32.52 | $24.41 | $27.66 | $27.66 | MBS item 105 |
| Hospital - Abortion W Gis | $2,290.60 |  |  | $2,290.60 | DRG O05Z Abortion W Gis |

1. Utilities Literature Search

A systematic review was identified from a PhD thesis^74^, which searched for studies eliciting utilities for pregnancy related health-states to inform economic evaluations. The systematic review was conducted in 2017. A supplementary search was conducted to update the 2017 review. This was undertaken by a member of the research team (AS), with a search span from 2017 to 2023. Quality assessment of the included studies, using the Yepes-Nunez items list for values and preference studies^75^ can be found in Table S8. It was pre-agreed we would choose the set of utilities based on study quality, applicability to our context, and the face validity of utility weights. We chose to use the utilities presented by Kupperman 2016^34^, as they covered the greatest number of health states (See Table S8 for values). The Kupperman study presents utilities for pathways, while we required utilities for discrete health states. As a result we anchored the utility of a live unaffected birth to one and calculated the other final health states accordingly. The face validity of the intermediate health states did not seem appropriate and as a result they were not included.

1. Quality assessment

Checklist items^10^

| **1. Was the instrument appropriately administered?** | | | | |  |  |  |  |  |
| --- | --- | --- | --- | --- | --- | --- | --- | --- | --- |
| Was the interviewer trained properly? | | |  |  |  |  |  |  |  |
| Was there a structured protocol for administering the instrument? | | | | | |  |  |  |  |
| Was a structured pre-testing procedure carried out? | | | | |  |  |  |  |  |
| Did investigators recruit optimal interviewees? | | | |  |  |  |  |  |  |
| Did the authors test for participant understanding? | | | |  |  |  |  |  |  |
| **2. Was the choice of the instrument optimal?** | | | |  |  |  |  |  |  |
| Did investigators use a standardized instrument with evidence of validity? | | | | | | |  |  |  |
| Was the questionnaire used adequately validated for their health condition? | | | | | | |  |  |  |
| If appropriate, did the authors present probabilities? | | | | |  |  |  |  |  |
| Were all relevant outcomes included? | | |  |  |  |  |  |  |  |
| Instrument(s): |  |  |  |  |  |  |  |  |  |
| Questionnaire(s): |  |  |  |  |  |  |  |  |  |
| **3. Was the choice of participants group optimal?** | | | |  |  |  |  |  |  |
| When patients were respondents, were their health states sufficiently similar? | | | | | | |  |  |  |
| Did the patients have previous experience of being involved in a choice related to the value and preference | | | | | | | | | |
|  | | | | | | | | | |
| choice under study? | |  |  |  |  |  |  |  |  |
| Were the participants representative of the underlying population? | | | | | |  |  |  |  |
| Was the sample appropriate according to the perspective of the study? | | | | | |  |  |  |  |
| Did the health state presentations have high verisimilitude/were appropriate? | | | | | | |  |  |  |
| Did the authors address the literature bearing on the patients’ views and experiences of the health conditions? | | | | | | | | | |
| Did the investigators justify their choice of health state: patients’ own vs hypothetical scenarios? | | | | | | | | |  |
| **5. Were the methods and results described, analyzed, and presented optimally?** | | | | | | |  |  |  |
| Were methods applied in the study clearly described? | | | | |  |  |  |  |  |
| Did the authors report the results clearly? | | | |  |  |  |  |  |  |
| Was the information presented convincingly? | | | |  |  |  |  |  |  |
| Did the authors adequately justify any omissions of data collected in the analyses? | | | | | | | |  |  |
| Did authors conduct the appropriate statistical analyses? | | | | |  |  |  |  |  |
| **6. Do results suggest patient understanding was adequate?** | | | | |  |  |  |  |  |
| Were the health states rated consistently? | | | |  |  |  |  |  |  |
| Did the authors address patient characteristics that may have a strong association with values and preferences? | | | | | | | | | |
| **7. Was a subgroup analysis conducted optimally?** | | | |  |  |  |  |  |  |
| Did the authors examine possible associations between patient characteristics and values and preferences? | | | | | | | | | |

**Table S8 Quality assessment for utility studies**

| **Author** | **Year** | **1** | **2** | **3** | **4** | **5** | **6** | **7** | **8** | **9** | **10** | **11** | **12** | **13** | **14** | **15** | **16** | **17** | **18** | **19** | **20** | **21** | **22** | **23** |
| --- | --- | --- | --- | --- | --- | --- | --- | --- | --- | --- | --- | --- | --- | --- | --- | --- | --- | --- | --- | --- | --- | --- | --- | --- |
| **Kupperman** | **2016** | ? | Y | ? | Y | ? |  |  | Y | Y | Y | ? | Y | Y | Y | Y | Y | Y | Y |  | Y | Y | Y | Y |
| **Deverill** | **2006** | ? | Y | ? | Y | ? |  |  | N | Y | N | ? | Y | Y | Y | Y | Y | Y | Y |  | Y | Y | Y |  |
| **Kupperman** | **2004** | ? | Y | ? | Y | ? |  |  | ? | Y | Y | ? | Y | Y | Y | Y | Y | Y | Y | Y | Y | Y | Y |  |
| **Grobman** | **2002** | Y | Y | Y | Y | ? |  |  | ? | ? | Y | ? | Y | Y | Y | Y | Y | Y | Y | Y | Y | Y | Y | Y |
| **Kupperman** | **2000** | Y | Y | ? | Y | Y |  |  | Y | Y | Y | ? | Y | Y | Y | Y | Y | Y | Y | Y | Y | Y | Y |  |
| **Verp** | **1995** | ? | ? | ? | N | ? |  |  | Y | ? | Y | ? | ? | ? | Y | Y | Y | Y | Y |  | Y |  |  |  |
| **Heckerling** | **1994** | Y | Y | ? | Y | Y |  |  | ? | ? | Y | ? | Y | Y | Y | Y | Y | Y | Y | Y | Y | Y | Y | Y |
| **Pauker** | **1987** | Y | Y | ? | Y | Y |  |  | Y | Y | Y | ? | Y | Y | Y | Y | Y | Y | Y | ? | Y | Y | Y | Y |

**
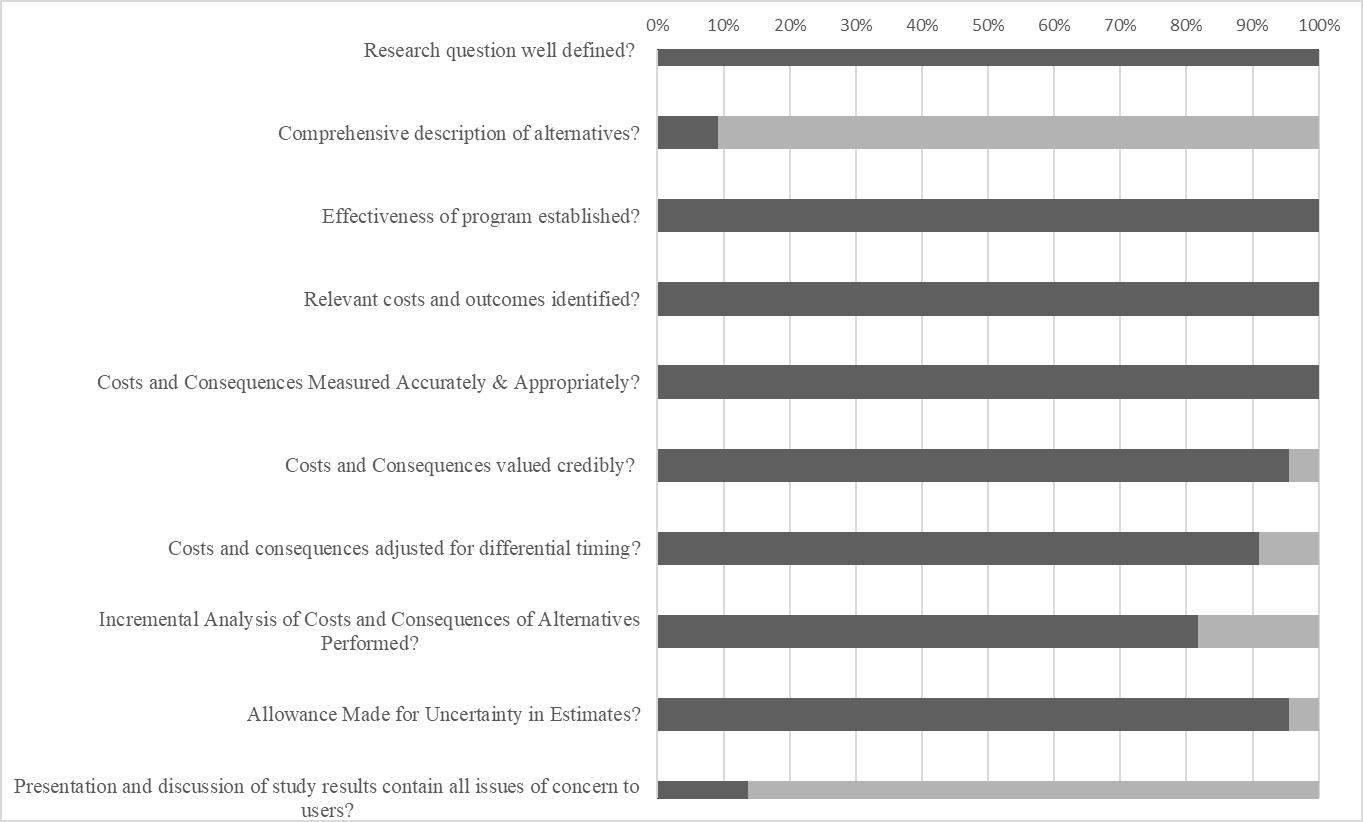
**

Figure 1 Quality assessment of included studies

1. Literature review results

**Table S9 Basic characteristics of included studies**

| **Study ID** | **Country** | **Study type** | **Perspective** | **Time horizon** | **Clinical condition** | **Cost discount rate** | **Outcome discount rate** | **Primary outcome measure(s)** | **Type of sensitivity analysis** |
| --- | --- | --- | --- | --- | --- | --- | --- | --- | --- |
| Anh (2021) | Vietnam | CUA | Societal | Lifetime | T21 | None | 3.0% | QALY of pregnant woman |  |
| Ayres (2015) | Australia | CEA | Healthcare funder + patient | Pregnancy to birth | T21 | None | None | T21 detected and PRL avoided |  |
| Bayón (2019) | Spain | CEA | healthcare funder | Pregnancy to birth | T21 | None | None | T21 detected |  |
| Benn (2015) | USA | Threshold analysis | Healthcare funder + patient | Lifetime | T21, T18, and T13 and monosomy X | None | None | Cases detected and PRL avoided |  |
| Beulen (2014) | The Netherlands | CEA | Healthcare funder + patient | Pregnancy to birth | T21 | None | None | T21 detected and PRL avoided |  |
| Colosi (2017) | Italy | Cost and consequence | Healthcare funder | Pregnancy to birth | T21, T18, and T13 | None | None | Cases detected and PRL avoided |  |
| Crimmins (2017) | USA | Threshold analysis | Healthcare funder + patient | Pregnancy to birth | T21 | None | None | T21 detected, PRL avoided and number of false positives |  |
| Cuckle (2013) | USA | CEA | healthcare funder | Pregnancy to birth | T21 | None | None | T21 detected |  |
| Fairbrother (2016) | USA | Threshold analysis | Healthcare funder + patient | Pregnancy to birth | T21, T18, and T13 | None | None | Cases detected |  |
| Garfield (2012) | USA | Cost and consequence | healthcare funder | Pregnancy to birth | T21, T18, and T13 | None | None | Cases detected and PRL avoided |  |
| Gyselaers (2015) | Belgium | Cost and consequence | healthcare funder | Pregnancy to birth | T21 | None | None | T21 detected |  |
| Hopkins (2020) | USA | CEA | healthcare funder | Pregnancy to birth | T21 | None | None | T21 detected, PRL avoided, number of unnecessary invasive tests |  |
| HQO (2019) | Canada | CEA | healthcare funder | Pregnancy to birth | T21, T18, T13, sex chromosome aneuploidies, and microdeletions | None | None | Cases detected |  |
| Huang (2020) | Canada | Cost and consequence | healthcare funder | Pregnancy to birth | T21 | None | None | T21 detected |  |
| IHE (2014) | Canada | CEA | healthcare funder | Pregnancy to birth | T21 | None | None | Cases detected and cases correctly diagnosed |  |
| Kaimal (2015) | USA | CUA | healthcare funder | Lifetime | T21, T18, and T13 | 3.0% | 3.0% | QALY of pregnant woman |  |
| KCE (2014) | Belgium | CEA | Healthcare funder + patient | Pregnancy to birth | T21 | None | None | T21 detected |  |
| Kostenko (2019) | Belgium | CEA | healthcare funder | Pregnancy to birth | T21, T18, and T13 | 5.0% | None | Cases detected |  |
| Maxwell (2017) | Australia | CEA | healthcare funder | Pregnancy to birth | T21 | None | None | T21 detected |  |
| Morris (2014) | UK | Cost and consequence | healthcare funder | Pregnancy to birth | T21 | None | None | T21 detected and PRL avoided |  |
| MSAC (2019) | Australia | CEA | Healthcare funder | Pregnancy to birth | T21, T18, and T13 | None | None | Cases detected |  |
| Neyt (2014) | Belgium | CEA | healthcare funder | Pregnancy to birth | T21 | None | None | T21 detected |  |
| Nshimyumukiza (2018) | Canada | CEA | healthcare funder | Pregnancy to birth | T21, T18, and T13 | None | None | T21 detected |  |
| Ökem (2017) | Turkey | CEA | Healthcare funder + patient | Pregnancy to birth | T21 | None | None | T21 detected and PRL avoided |  |
| Okun (2014) | Canada | CEA | healthcare funder | Pregnancy to birth | T21 | None | None | T21 detected |  |
| O'Leary (2013) | Australia | CEA | healthcare funder | Pregnancy to birth | T21 | None | None | T21 detected |  |
| Shang (2021) | China | CEA | healthcare funder | Pregnancy to birth | T21 | None | None | T21 averted |  |
| Song (2013) | USA | CEA | Societal | First 5 years of life | T21 | 3.0% | None | T21 detected |  |
| Walker (2014) | USA | CEA | Societal | Lifetime | T21 | None | None | T21 detected |  |
| Walker (2015) | USA | CEA | Societal and funder | Lifetime | T21, T18, and T13 | 3.0% | None | Cases detected |  |
| Wanapirak (2019)^51^ | Thailand | CBA | Societal and funder | Lifetime | T21 | None | None | T21 averted |  |
| Xie (2020) | Canada | CEA | healthcare funder | Pregnancy to birth | T21 | None | None | T21 detected |  |
| Xu (2019) | China | CEA | Societal | Pregnancy to birth | T21, T18, T13, sex chromosome aneuploidies, and microdeletions | None | None | T21 averted |  |
| Zhang (2019) | Canada | CUA | Healthcare funder + patient | Pregnancy until 18yrs old | T21 | 3.0% | 3.0% | QALY of pregnant woman |  |

Abbreviations: CBA, cost-benefit analysis; CEA, cost-effectiveness analysis; CUA, cost-utility analysis; PRL, procedure-related loss; QALY, quality adjusted life year.

**Table S 10 Identified cost utility analyses and utility weights**

| **Pregnancy scenario** | **Kaimal 2015^20^** | | | | **Zhang 2019^21^** | | | | **Anh 2021^45^** | | | |
| --- | --- | --- | --- | --- | --- | --- | --- | --- | --- | --- | --- | --- |
|  | **Mean utility** | **(SD)** | **Source** | **Method** | **Mean utility** | **(SD)** | **Source** | **Method** | **Mean utility** | **CI** | **Source** | **Method** |
| ***One screening test with low-risk results*** | | | | | | | | | | | | |
| Unspecified screening test, low-risk result | 0.931 | 0.154 | Kaimal 2015**^20^** | TTO | 0.931 | 0.154 | Kupperman 2016 | TTO | NR | NR | NR | NR |
| ***Diagnostic testing with normal results*** | | | | | | | | | | | | |
| cfDNA, no result; CVS or amniocentesis, normal result | 0.928 | 0.15 | Kaimal 2015**^20^** | TTO | 0.921 | 0.161 | Kupperman 2016 | TTO | 0.923 | 0.62;1 | Harris 2004 | TTO |
| MMS, increased risk result; CVS or amniocentesis, normal result | 0.925 | 0.161 | Kaimal 2015**^20^** | TTO | 0.921 | 0.161 | Kupperman 2016 | TTO | 0.923 | 0.62;1 | Harris 2004 | TTO |
| Straight to CVS or amniocentesis (with no prior screening), normal result | 0.921 | 0.161 | Kaimal 2015**^20^** | TTO | 0.921 | 0.161 | Kupperman 2016 | TTO | 0.923 | 0.62;1 | Harris 2004 | TTO |
| ***Two screening tests with differing results*** | | | | | | | | | | | | |
| MMS alone, increased risk result; cfDNA, low-risk result | 0.89 | 0.199 | Kaimal 2015**^20^** | TTO | NR | NR | NR | NR | NR | NR | NR | NR |
| MMS and cfDNA together, MMS increased risk result and cfDNA low-risk result | 0.85 | 0.229 | Kaimal 2015**^20^** | TTO | NR | NR | NR | NR | NR | NR | NR | NR |
| ***Pregnancy termination in the context of abnormal results*** | | | | | | | | | | | | |
| One unspecified screening test, increased risk result; CVS or amniocentesis, abnormal result; terminate | 0.771 | 0.284 | Kaimal 2015 unpublished | TTO | 0.771 | 0.284 | Kupperman 2016^34^ | TTO | 0.836 | 0.4,1.0 | Harris 2004 | TTO |
| MMS alone, increased risk result; cfDNA test, increased risk result; CVS or amniocentesis, abnormal result; terminate | 0.771 | 0.268 | Kaimal 2015 unpublished | TTO | 0.771 | 0.284 | Kupperman 2016^34^ | TTO | 0.836 | 0.4,1.0 | Harris 2004 | TTO |
| Straight to CVS or amniocentesis, abnormal result; terminate | 0.725 | 0.294 | Kaimal 2015 unpublished | TTO | NR | NR | NR | NR | 0.836 | 0.4,1.0 | Harris 2004 | TTO |
| *Pregnancy termination in the context of VUS results* |  |  |  |  |  |  |  |  |  |  |  |  |
| Unspecified screening test, increased risk result; CVS or amniocentesis, VUS; terminate | 0.762 | 0.29 | Kaimal 2015 unpublished | TTO | NR | NR | NR | NR | 0.836 | 0.4,1.0 | Harris 2004 | TTO |
| Straight to CVS or amniocentesis, VUS; terminate | 0.694 | 0.3 | Kaimal 2015 unpublished | TTO | NR | NR | NR | NR | 0.836 | 0.4,1.0 | Harris 2004 | TTO |
| ***Miscarriage*** | | | | | | | | | | | | |
| Unspecified screening test, increased risk result; CVS or amniocentesis, miscarriage | 0.744 | 0.285 | Kaimal 2015 unpublished | TTO | NR | NR | NR | NR | 0.76 | 0.68;0.94 | Kuppermann 2000 | TTO |
| Straight to CVS or amniocentesis, procedure related miscarriage | 0.663 | 0.28 | Kaimal 2015 unpublished | TTO | NR | NR | NR | NR | 0.76 | 0.68;0.94 | Kuppermann 2000 | TTO |
| Spontaneous loss (affected) | NR |  |  |  | 0.744 | 0.285 | Kupperman 2016 | TTO | 0.76 | 0.68;0.94 | Kuppermann 2000 | TTO |
| Spontaneous loss (unaffected) | NR |  |  |  | 0.744 | 0.285 | Kupperman 2016 | TTO | 0.76 | 0.68;0.94 | Kuppermann 2000 | TTO |
| ***Pregnancy continuation in the context of screen positive results with no further testing or abnormal diagnostic test results*** | | | | | | | | | | | | |
| cfDNA, no result; no further testing, continue pregnancy | 0.763 | 0.281 | Kaimal 2015 unpublished | TTO | NR | NR | NR | NR | NR | NR | NR | NR |
| MMS, increased risk result; no further testing, continue pregnancy | 0.756 | 0.258 | Kaimal 2015 unpublished | TTO | NR | NR | NR | NR | NR | NR | NR | NR |
| MMS, increased risk result; cfDNA, increased risk result; no further testing, continue pregnancy | 0.682 | 0.27 | Kaimal 2015 unpublished | TTO | NR | NR | NR | NR | 0.69 | 0.54;0.88 | Kuppermann 2000 | TTO |
| Unspecified screening test, increased risk result; CVS or amniocentesis, abnormal result; continue pregnancy | 0.655 | 0.282 | Kaimal 2015 unpublished | TTO | NR | NR | NR | NR | 0.69 | 0.54;0.88 | Kuppermann 2000 | TTO |
| ***Pregnancy continuation in the context of VUS*** | | | | | | | | | | | | |
| Unspecified screening test, increased risk result; CVS or amniocentesis, VUS; continue pregnancy | 0.806 | 0.234 | Kaimal 2015 unpublished | TTO | NR | NR | NR | NR | NR | NR | NR | NR |
| ***Long term outcomes*** | | | | | | | | | | | | |
| Baby with Down syndrome or another intellectual disability | 0.48 | 0.305 | Kaimal 2015 unpublished | TTO | 0.48 | 0.305 | Kupperman 2016 | TTO | 0.55 | 0.28;0.82 | Mok 2014 | Multi attribute questionnaire - HUI3 |
| Baby with trisomy 13 or 18 | 0.495 | 0.323 | Kaimal 2015 unpublished | TTO | NR | NR | NR | NR | NR | NR | NR | NR |
| Baby with VUS | 0.737 | 0.28 | Kaimal 2015 unpublished | TTO | NR | NR | NR | NR | NR | NR | NR | NR |
| Pregnancy loss with repeat pregnancy and birth of a healthy baby 2 years later | 0.88 | 0.178 | Kaimal 2015 unpublished | TTO | 0.88 | 0.178 | Kupperman 2016 | TTO | NR | NR | NR | NR |
| Pregnancy loss with no future pregnancy | 0.59 | 0.313 | Kaimal 2015 unpublished | TTO | 0.59 | 0.313 | Kupperman 2016 | TTO | NR | NR | NR | NR |

Abbreviations: TTO, time trade off; NR, not reported.

1. Sensitivity analysis results

**Table S 11a One-way sensitivity analysis: Models D3 and D5 (AUD)**

|  | **Base case** | **Range** | **Source** | **Model D3** | | | | | | **Model D5** | | | | | |
| --- | --- | --- | --- | --- | --- | --- | --- | --- | --- | --- | --- | --- | --- | --- | --- |
|  |  |  |  | **Cost/T21 (AUD)** | | **Cost/PRL avoided (AUD)** | | **Cost/QALY (AUD)** | | **Cost/T21 (AUD)** | | **Cost/PRL avoided (AUD)** | | **Cost/QALY (AUD)** | |
|  |  |  |  | **Low value** | **High value** | **Low value** | **High value** | **Low value** | **High value** | **Low value** | **High value** | **Low value** | **High value** | **Low value** | **High value** |
| NIPT sensitivity | 100% | 90.7% to 100% | Norton 2015^11^ | -8,181 | 93,141 | 11,599 | 12,527 | 46,158 | 49,590 | -6,644 | 96,873 | 11,694 | 12,855 | 20,546 | 21,188 |
| NIPT specificity | 99.90% | 99.9% to 100% | Norton 2015^11^ | 93,141 | 91,600 | 12,527 | 12,307 | 49,590 | 48,731 | 96,873 | 95,332 | 12,855 | 12,635 | 21,188 | 20,842 |
| cFTS sensitivity | 78.90% | 62.7% to 90.4% | Norton 2015^11^ | 97,991 | 90,721 | 10,566 | 13,919 | 41,781 | 55,132 | 101,852 | 94,389 | 10,848 | 14,280 | 20,347 | 21,670 |
| cFTS specificity | 94.60% | 94.2% to 94.9% | Norton 2015^11^ | 94,268 | 92,296 | 11,765 | 13,181 | 46,561 | 52,189 | 98,045 | 95,994 | 10,173 | 13,524 | 20,792 | 21,501 |
| Uptake of cFTS | 83.60% | 60% to 100% | Maxwell 2011^88^ | 93,131 | 93,141 | 12,527 | 12,527 | 49,590 | 49,590 | 96,873 | 96,873 | 12,855 | 12,855 | 21,188 | 21,188 |
| Uptake of NIPT | 100% | 76% to 100% | HQO 2019^13^ | 9,692 | 93,141 | -38,371 | 12,527 | -151,633 | 49,590 | 11,351 | - | - | 12,855 | -217,023 | 21,188 |
| Uptake of diagnostic testing after cFTS | 80% | 40% to 100% | Lindquist 2019^73^ | 13,498 | 9,666 | 275,459 | -31,561 | 11,001,646 | -124,281 | 15,238 | 11,311 | 310,556 | -37,062 | 117,091 | -135,398 |
| Uptake of diagnostic testing after high cFTS | 90% | 40% to 100% | Lindquist 2019^73^ | 66,804 | 97,290 | 7,137 | 13,641 | 28,529 | 53,943 | 71,014 | 100,946 | 7,418 | 13,979 | 12,589 | 22,922 |
| Uptake of diagnostic testing after NIPT | 80% | 40% to 100% | Lindquist 2019 ^73^ | -985 | 3,407 | 7,932 | 14,907 | 32,248 | 58,163 | 708 | 5,196 | -5,781 | 22,508 | 27,108 | 21,806 |

Abbreviations: cFTS, combined first trimester screening; NIPT, non-invasive prenatal testing; PRL, procedure-related loss; QALY, Quality adjusted life year.

**Table S 12b One-way sensitivity analysis: Models D3 and D5 (Converted to USD)**

|  | **Base case** | **Range** | **Source** | **Model D3** | | | | | | **Model D5** | | | | | |
| --- | --- | --- | --- | --- | --- | --- | --- | --- | --- | --- | --- | --- | --- | --- | --- |
|  |  |  |  | **Cost/T21** | | **Cost/PRL avoided** | | **Cost/QALY** | | **Cost/T21** | | **Cost/PRL avoided** | | **Cost/QALY** | |
|  |  |  |  | **Low value** | **High value** | **Low value** | **High value** | **Low value** | **High value** | **Low value** | **High value** | **Low value** | **High value** | **Low value** | **High value** |
| NIPT sensitivity | 100% | 90.7% to 100% | Norton 2015^11^ | -5,764 | 65,626 | 8,173 | 8,826 | 32,523 | 34,941 | 4,681 | 68,256 | 8,240 | 9,058 | 14477 | 14929 |
| NIPT specificity | 99.90% | 99.9% to 100% | Norton 2015^11^ | 65,626 | 64,541 | 8,826 | 8,671 | 34,941 | 34,335 | 68,256 | 67,170 | 9,058 | 8,903 | 14929 | 14685 |
| cFTS sensitivity | 78.90% | 62.7% to 90.4% | Norton 2015^11^ | 69,044 | 63,921 | 7,445 | 9,807 | 29,439 | 38,846 | 71,764 | 66,506 | 7,643 | 10,062 | 14336 | 15269 |
| cFTS specificity | 94.60% | 94.2% to 94.9% | Norton 2015^11^ | 66,421 | 65,031 | 8,290 | 9,287 | 32,807 | 36,772 | 69,082 | 67,637 | 7,168 | 9,529 | 14650 | 15149 |
| Uptake of cFTS | 83.60% | 60% to 100% | Maxwell 2011^88^ | 65,619 | 65,626 | 8,826 | 8,826 | 34,941 | 34,941 | 68,256 | 68,256 | 9,058 | 9,058 | 14929 | 14929 |
| Uptake of NIPT | 100% | 76% to 100% | HQO 2019^13^ | 6,829 | 65,626 | -27,036 | 8,826 | -106,839 | 34,941 | 7,998 | - | - | 9,058 | -152913 | 14929 |
| Uptake of diagnostic testing after cFTS | 80% | 40% to 100% | Lindquist 2019^73^ | 9,511 | 6,811 | 194,086 | -22,238 | 7,751,678 | -87,567 | 10,737 | 7,970 | 218,815 | 26,114 | 82501 | -95400 |
| Uptake of diagnostic testing after high cFTS | 90% | 40% to 100% | Lindquist 2019^73^ | 47,070 | 68,550 | 5,029 | 9,611 | 20,101 | 38,008 | 50,036 | 71,126 | 5,227 | 9,849 | 8870 | 16151 |
| Uptake of diagnostic testing after NIPT | 80% | 40% to 100% | Lindquist 2019 ^73^ | -694 | 2,401 | 5,589 | 10,503 | 22,722 | 40,981 | 499 | 3,661 | -4,073 | 15,859 | 19100 | 15364 |

Abbreviations: cFTS, combined first trimester screening; NIPT, non-invasive prenatal testing; PRL, procedure-related loss; QALY, Quality adjusted life year.

**Table S13a One-way sensitivity analysis: Models M3 and M5 (AUD)**

|  | **Base case** | **Range** | **Source** | **Model M3** | | | | | | **Model M5** | | | | | |
| --- | --- | --- | --- | --- | --- | --- | --- | --- | --- | --- | --- | --- | --- | --- | --- |
|  |  |  |  | **Cost/T21** | | **Cost/PRL avoided** | | **Cost/QALY** | | **Cost/T21** | | **Cost/PRL avoided** | | **Cost/QALY** | |
|  |  |  |  | **Low value** | **High value** | **Low value** | **High value** | **Low value** | **High value** | **Low value** | **High value** | **Low value** | **High value** | **Low value** | **High value** |
| NIPT sensitivity | 100% | 90.7% to 100% | Norton 2015^11^ | -9,196 | -139,361 | 17,192 | 18,177 | 76,073 | 80,431 | -8,346 | -156,700 | -15,637 | -20,439 | -15,547 | -22,676 |
| NIPT specificity | 99.90% | 99.9% to 100% | Norton 2015^11^ | -139,361 | -139,361 | 18,177 | 17,959 | 80,431 | 79,465 | -156,700 | -155,026 | -20,439 | -20,220 | -22,676 | -22,433 |
| cFTS sensitivity | 78.90% | 62.7% to 90.4% | Norton 2015^11^ | -61,377 | -152,657 | 20,495 | 20,576 | 70,846 | 88,105 | -70,046 | -166,744 | -22,191 | -22,413 | -25,684 | -20,211 |
| cFTS specificity | 94.60% | 94.2% to 94.9% | Norton 2015^11^ | -143,055 | -132,124 | 18,177 | 18,017 | 75,160 | 80,205 | -163,644 | -149,463 | -19,636 | -20,381 | -23,392 | -21,365 |
| Uptake of cFTS | 83.60% | 60% to 100% | Maxwell 2011^88^ | -286,242 | -126,617 | 16,838 | 18,758 | 78,166 | 86,178 | -299,246 | -146,122 | -17,602 | -21,647 | -21,657 | -21,396 |
| Uptake of NIPT | 100% | 76% to 100% | HQO 2019^13^ | 8,657 | -139,362 | -41,401 | 18,177 | - 183,191 | 80,432 | 9,356 | -156,700 | 44,739 | -20,439 | 37,063 | -22,676 |
| Uptake of diagnostic testing after cFTS | 80% | 40% to 100% | Lindquist 2019^73^ | 3,339 | 8,986 | 73,458 | -33,699 | 1,203,335 | 143,742 | 15,308 | 10,205 | -313,823 | 38,270 | 1,123,474 | 44,839 |
| Uptake of diagnostic testing after high cFTS | 90% | 40% to 100% | Lindquist 2019^73^ | -94,519 | 150,151 | 12,328 | 19,585 | 54,551 | 86,659 | -124,860 | 166,405 | -16,286 | -21,705 | -15,736 | -24,381 |
| Uptake of diagnostic testing after NIPT | 80% | 40% to 100% | Lindquist 2019^73^ | -1,269 | 4,145 | 12,916 | 22,042 | 57,151 | 91,721 | 272 | 6,339 | 2,773 | -33,716 | 1,991 | -49,335 |

Abbreviations: cFTS, combined first trimester screening; NIPT, non-invasive prenatal testing; PRL, procedure-related loss; QALY, Quality adjusted life year.

**Table S14 One-way sensitivity analysis: Models 3 and 4 (converted to USD)**

|  | **Base case** | **Range** | **Source** | **Model 3** | | | | | | **Model 4** | | | | | |
| --- | --- | --- | --- | --- | --- | --- | --- | --- | --- | --- | --- | --- | --- | --- | --- |
|  |  |  |  | **Cost/T21** | | **Cost/PRL avoided** | | **Cost/QALY** | | **Cost/T21** | | **Cost/PRL avoided** | | **Cost/QALY** | |
|  |  |  |  | **Low value** | **High value** | **Low value** | **High value** | **Low value** | **High value** | **Low value** | **High value** | **Low value** | **High value** | **Low value** | **High value** |
| NIPT sensitivity | 100% | 90.7% to 100% | Norton 2015^11^ | -6,479 | -98,193 | 12,113 | 12,807 | 53,600 | 56,671 | -5,881 | -110,410 | -11,018 | -14,401 | -10,954 | -15,977 |
| NIPT specificity | 99.90% | 99.9% to 100% | Norton 2015^11^ | -98,193 | -98,193 | 12,807 | 12,654 | 56,671 | 55,990 | -110,410 | -109,230 | -14,401 | -14,247 | -15,977 | -15,806 |
| cFTS sensitivity | 78.90% | 62.7% to 90.4% | Norton 2015^11^ | -43,246 | -107,561 | 14,441 | 14,498 | 49,918 | 62,078 | -49,354 | -117,487 | -15,636 | -15,792 | -18,097 | -14,241 |
| cFTS specificity | 94.60% | 94.2% to 94.9% | Norton 2015^11^ | -100,795 | -93,094 | 12,807 | 12,695 | 52,957 | 56,512 | -115,302 | -105,311 | -13,835 | -14,360 | -16,482 | -15,054 |
| Uptake of cFTS | 83.60% | 60% to 100% | Maxwell 2011^88^ | -201,684 | -89,213 | 11,864 | 13,217 | 55,075 | 60,720 | -210,846 | -102,956 | -12,402 | -15,252 | -15,259 | -15,075 |
| Uptake of NIPT | 100% | 76% to 100% | HQO 2019^13^ | 6,100 | -98,193 | -29,171 | 12,807 | -129,075 | 56,672 | 6,592 | -110,410 | 31,523 | -14,401 | 26,114 | -15,977 |
| Uptake of diagnostic testing after cFTS | 80% | 40% to 100% | Lindquist 2019^73^ | 2,353 | 6,331 | 51,758 | -23,744 | 847,861 | 101,280 | 10,786 | 7,190 | -221,117 | 26,965 | 791,591 | 31,593 |
| Uptake of diagnostic testing after high cFTS | 90% | 40% to 100% | Lindquist 2019^73^ | -66,597 | 105,795 | 8,686 | 13,799 | 38,436 | 61,059 | -87,975 | 117,248 | -11,475 | -15,293 | -11,087 | -17,179 |
| Uptake of diagnostic testing after NIPT | 80% | 40% to 100% | Lindquist 2019^73^ | -894 | 2,921 | 9,101 | 15,531 | 40,268 | 64,626 | 192 | 4,466 | 1,954 | -23,756 | 1,403 | -34,761 |

Abbreviations: cFTS, combined first trimester screening; NIPT, non-invasive prenatal testing; PRL, procedure-related loss; QALY, Quality adjusted life year

1. Probabilistic Sensitivity Analysis

**Table S 15 Probabilistic sensitivity analysis**

| **Threshold** | **Model D3** | | | **Model D5** | | |
| --- | --- | --- | --- | --- | --- | --- |
|  | **T21** | **PRL avoided** | **QALY** | **T21** | **PRL avoided** | **QALY** |
| 25,000 AUD 2023/outcome  *(17,614 USD 2023)* | NIPT will be funded in 17.8% of cases | NIPT will be funded in 74.6% of cases | NIPT will be funded in 42.5% of cases | NIPT will be funded in 23.9% of cases | NIPT will be funded in 84.4% of cases | NIPT will be funded in 57% of cases |
| 50,000 AUD 2023/outcome *(35,300 USD 2023)* | cFTS will be funded in 21.6% of cases | NIPT funded in 93% of cases | NIPT will be funded in 44.6% of cases | NIPT will be funded in 31.7% of cases | NIPT will be funded in 95% of cases | NIPT will be funded in 76% of cases |
| 100,000 AUD 2023/outcome *(70,459 USD 2023)* | cFTS will be funded in 23.4% of cases | NIPT funded in 99.5% of cases | NIPT will be funded in 75.6% of cases | NIPT will be funded in 35%of cases | NIPT will be funded in 99.6% of cases | NIPT will be funded in 94% of cases |

Abbreviations: cFTS, combined first trimester screening; NIPT, non-invasive prenatal testing; PRL, procedure-related loss; QALY, Quality adjusted life year.

**Table S 16 Probabilistic sensitivity analysis**

| **Threshold** | **Model M3** | | | **Model M5** | | |
| --- | --- | --- | --- | --- | --- | --- |
|  | **T21** | **PRL avoided** | **QALY** | **T21** | **PRL avoided** | **QALY** |
| 25,000 AUD 2023/outcome  *(17,614 USD 2023)* | NIPT will be funded in 0% of cases | NIPT will be funded in 96% of cases | NIPT will be funded in 0% of cases | NIPT will be funded in 0% of cases | NIPT will be funded in 95% of cases | NIPT will be funded in 0% of cases |
| 50,000 AUD 2023/outcome *(35,300 USD 2023)* | NIPT will be funded in 0% of cases | NIPT will be funded in 100% of cases | NIPT will be funded in 74% of cases | NIPT will be funded in 0% of cases | NIPT will be funded in 100% of cases | NIPT will be funded in 0% of cases |
| 100,000 AUD 2023/outcome *(70,459 USD 2023)* | NIPT will be funded in 26% of cases | NIPT will be funded in 100% of cases | NIPT will be funded in 95% of cases | NIPT will be funded in 26% of cases | NIPT will be funded in 100% of cases | NIPT will be funded in 0% of cases |

Abbreviations: cFTS, combined first trimester screening; NIPT, non-invasive prenatal testing; PRL, procedure-related loss; QALY, Quality adjusted life year.

1. Model Structures


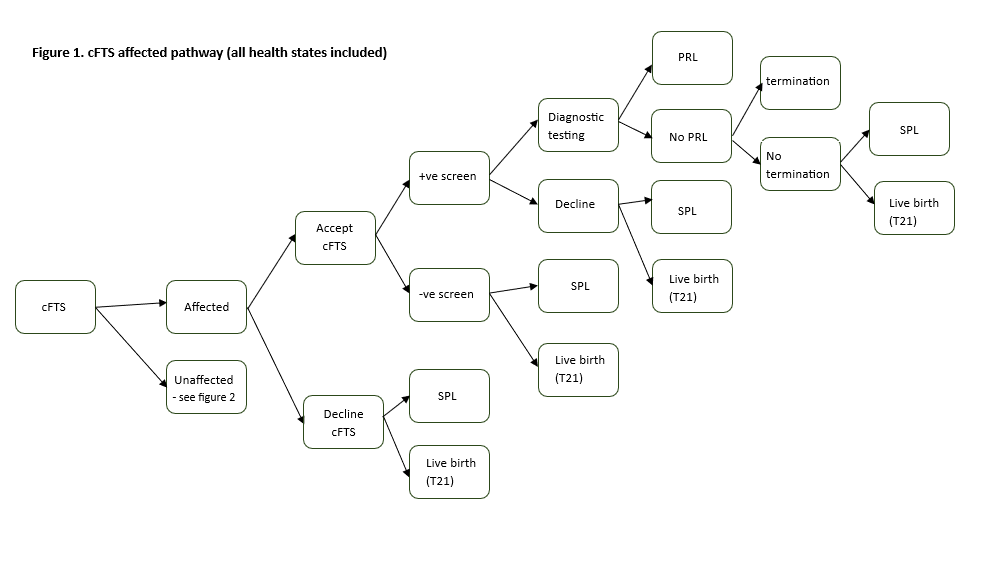


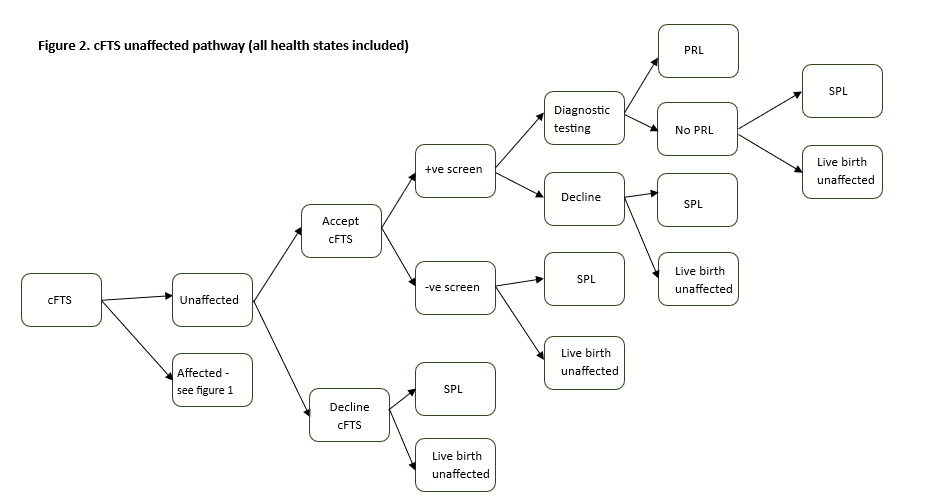


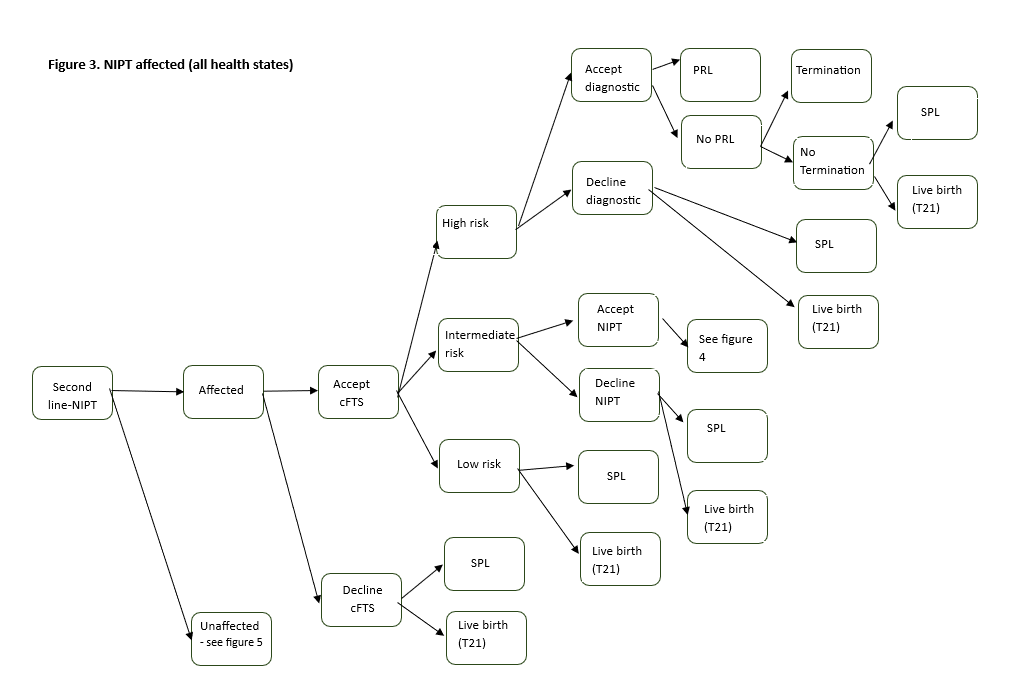


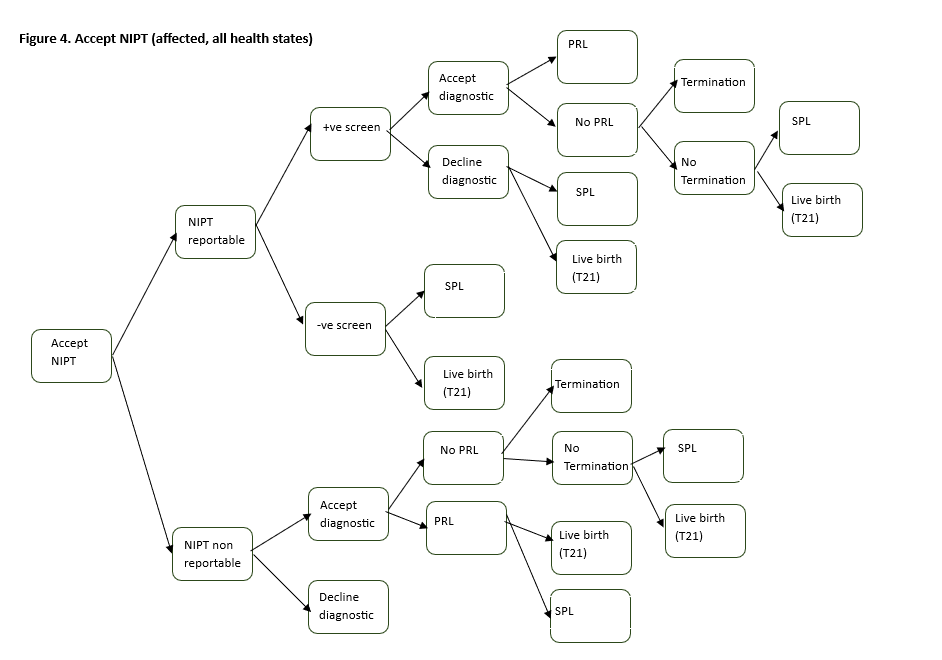


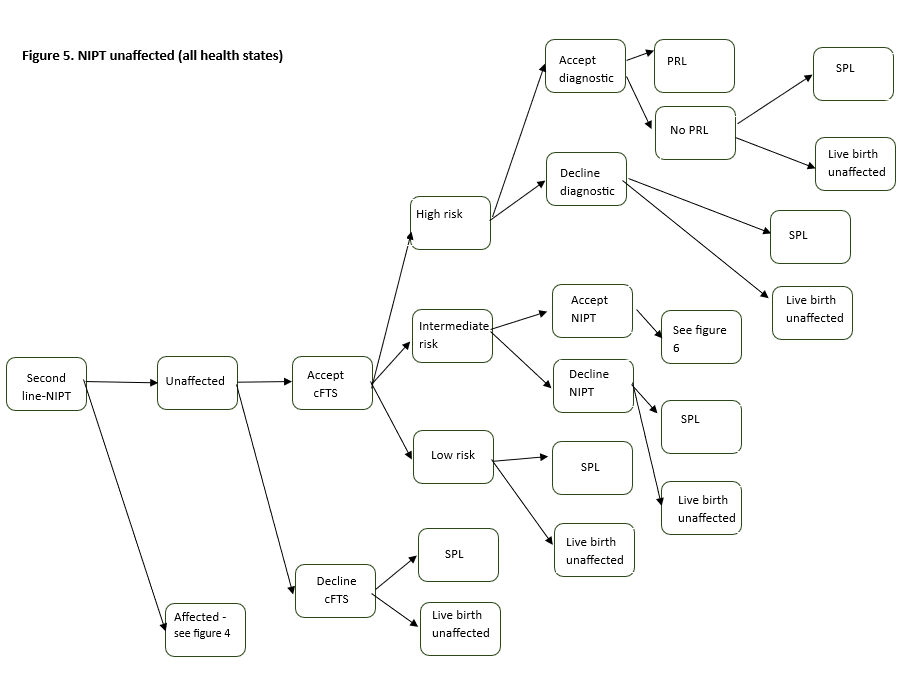


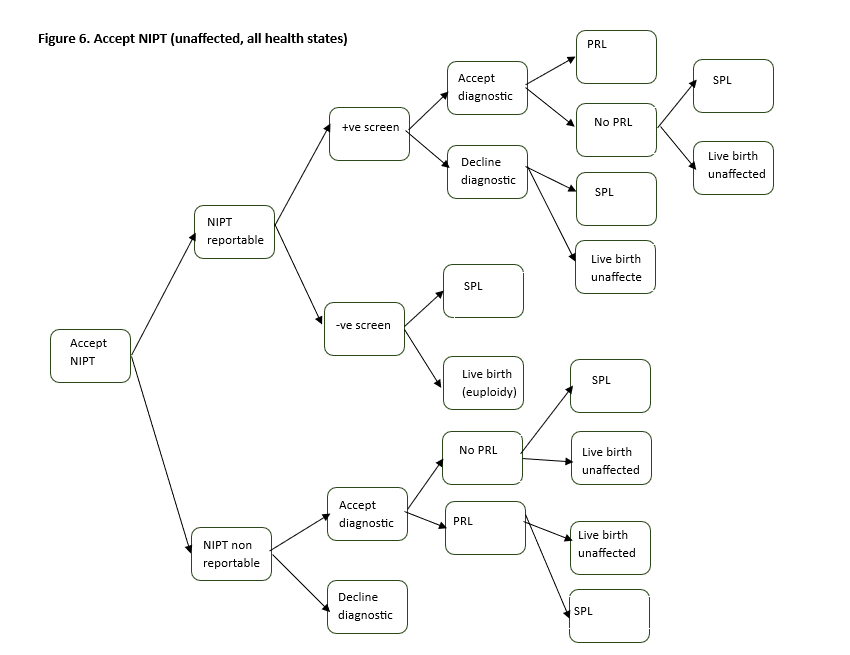


**Supplementary Materials References**

1. Maxwell S, Bower C, O'Leary P. Impact of prenatal screening and diagnostic testing on trends in Down syndrome births and terminations in Western Australia 1980 to 2013: Impact of prenatal screening and diagnosis on Down syndrome, 1980-2013. Prenatal diagnosis. 2015; 35: 1324-30.

2. Ammon Avalos L, Galindo C, Li D-K. A systematic review to calculate background miscarriage rates using life table analysis. Birth defects research A Clinical and molecular teratology. 2012; 94: 417-23.

3. Morris JK, Wald NJ, Watt HC. Fetal loss in Down syndrome pregnancies. Prenatal diagnosis. 1999; 19: 142-45.

4. Norton ME, Jacobsson B, Swamy GK, et al. Cell-free DNA Analysis for Noninvasive Examination of Trisomy. The New England journal of medicine. 2015; 372: 1589-97.

5. Salomon LJ, Sotiriadis A, Wulff CB, Odibo A, Akolekar R. Risk of miscarriage following amniocentesis or chorionic villus sampling: systematic review of literature and updated meta‐analysis. Ultrasound in obstetrics & gynecology. 2019; 54: 442-51.

6. Maxwell S, O'Leary P, Dickinson JE, Suthers GK. Diagnostic performance and costs of contingent screening models for trisomy 21 incorporating non-invasive prenatal testing. Australian and New Zealand Journal of Obstetrics and Gynaecology. 2017; 57: 432-39.

7. Maxwell S, James I, Dickinson JE, O'Leary P. First trimester screening cut-offs for noninvasive prenatal testing as a contingent screen: Balancing detection and screen-positive rates for trisomy 21. Australian & New Zealand journal of obstetrics & gynaecology. 2016; 56: 29-35.

8. Lindquist A, Hui L, Poulton A, et al. State‐wide utilization and performance of traditional and cell‐free DNA‐based prenatal testing pathways: the Victorian Perinatal Record Linkage (PeRL) study. Ultrasound in obstetrics & gynecology. 2020; 56: 215-24.

9. MSAC. Public Summary Document. Application No. 1492 – Non-invasive prenatal testing (NIPT) for trisomies 21, 18 and 13. Australia: Medical Services Advisory Committee, 2019.

10. D'Souza R. Exploring Methodologic Challenges in the Conduct of Patient-Preference Studies in Obstetrics. University of Toronto (Canada), 2019.

11. Yepes-Nuñez JJ, Zhang Y, Xie F, et al. Forty-two systematic reviews generated 23 items for assessing the risk of bias in values and preferences' studies. Journal of clinical epidemiology. 2017; 85: 21-31.

12. Kuppermann M, Norton ME, Thao K, et al. Preferences regarding contemporary prenatal genetic tests among women desiring testing: implications for optimal testing strategies: Preferences, inclinations, and optimal prenatal testing decision-making. Prenatal diagnosis. 2016; 36: 469-75.

13. Deverill M, Robson S. Women's preferences in screening for Down syndrome. Prenatal diagnosis. 2006; 26: 837-41.

14. Kuppermann M, Nease Jr RF, Gates E, et al. How do women of diverse backgrounds value prenatal testing outcomes? Prenatal diagnosis. 2004; 24: 424-29.

15. Grobman WA, Dooley SL, Welshman EE, Pergament E, Calhoun EA. Preference assessment of prenatal diagnosis for Down syndrome: is 35 years a rational cutoff? Prenatal diagnosis. 2002; 22: 1195-200.

16. Kuppermann M, Nease RF, Learman LA, et al. Procedure-related miscarriages and down syndrome–affected births: implications for prenatal testing based on women’s preferences. Obstetrics and gynecology (New York 1953). 2000; 96: 511-16.

17. Verp MS, Heckerling PS. Use of decision analysis to evaluate patients' choices of diagnostic prenatal test. American journal of medical genetics. 1995; 58: 337-44.

18. Heckerling PS, Verp MS, Hadro TA. Preferences of pregnant women for amniocentesis or chorionic villus sampling for prenatal testing: Comparison of patients' choices and those of a decision-analytic model. Journal of clinical epidemiology. 1994; 47: 1215-28.

19. Pauker SP, Pauker SG. The amniocentesis decision: ten years of decision analytic experience. Birth Defects Original Article Series. 1987; 23: 151-69.

20. Anh ND, Trang LDM, Anh NQ. First-trimester screening versus non-invasive prenatal testing for Down syndrome at high-risk pregnant women in Hanoi Obstetrics and Gynecology Hospital, Vietnam: A cost-utility analysis. International Journal of Healthcare Management. 2021; 14: 1318-25.

21. Ayres AC, Whitty JA, Ellwood DA. A cost-effectiveness analysis comparing different strategies to implement noninvasive prenatal testing into a down syndrome screening program. Obstetrical and Gynecological Survey. 2015; 70: 63-65.

22. Bayón JC, Orruño E, Portillo MI, Asua J. The consequences of implementing non-invasive prenatal testing with cell-free foetal DNA for the detection of Down syndrome in the Spanish National Health Service: A cost-effectiveness analysis. Cost Effectiveness and Resource Allocation. 2019; 17.

23. Benn P, Curnow KJ, Chapman S, et al. An economic analysis of cell-free DNA Non-invasive prenatal testing in the US general pregnancy population. PLoS ONE. 2015; 10.

24. Beulen L, Grutters JPC, Faas BH, et al. The consequences of implementing non-invasive prenatal testing in Dutch National Health Care: A cost-effectiveness analysis: Editorial comment. Obstetrical and Gynecological Survey. 2015; 70: 162-64.

25. Colosi E, D'Ambrosio V, Periti E. First trimester contingent screening for trisomies 21,18,13: is this model cost efficient and feasible in public health system? The journal of maternal-fetal & neonatal medicine. 2017; 30: 2905-10.

26. Crimmins S, Doyle L, Slejko J, Kopelman JN, Turan O. QUAD versus cfDNA in an urban population in the second trimester for detection of trisomy 21: a cost sensitivity analysis. Journal of Maternal-Fetal and Neonatal Medicine. 2017; 30: 2334-39.

27. Cuckle H, Benn P, Pergament E. Maternal cfDNA screening for Down syndrome - a cost sensitivity analysis. Prenatal diagnosis. 2013; 33: 636-42.

28. Fairbrother G, Burigo J, Sharon T, Song K. Prenatal screening for fetal aneuploidies with cell-free DNA in the general pregnancy population: A cost-effectiveness analysis. Journal of Maternal-Fetal and Neonatal Medicine. 2016; 29: 1160-64.

29. Garfield SS, Armstrong SO. Clinical and cost consequences of incorporating a novel non-invasive prenatal test into the diagnostic pathway for fetal trisomies. Journal of Managed Care Medicine. 2012; 15: 32-39.

30. Gyselaers W, Hulstaert F, Neyt M. Contingent non-invasive prenatal testing: an opportunity to improve non-genetic aspects of fetal aneuploidy screening. Prenatal diagnosis. 2015; 35: 1347-52.

31. Hopkins MK, Dugoff L, Durnwald C, Havrilesky LJ, Dotters-Katz S. Cell-free DNA for Down syndrome screening in obese women: Is it a cost-effective strategy? Prenatal diagnosis. 2020; 40: 173-78.

32. Anonymous. Ontario health technology assessment series: Noninvasive prenatal testing for trisomies 21, 18, and 13, sex chromosome aneuploidies, and microdeletions: A health technology assessment. Ontario Health Technology Assessment Series. 2019; 19: 1-166.

33. Huang T, Gibbons C, Rashid S, et al. Prenatal screening for trisomy 21: a comparative performance and cost analysis of different screening strategies. BMC Pregnancy and Childbirth. 2020; 20.

34. IHE. First and second trimester prenatal screening update. . Institute of Health Econoimcs, 2014.

35. Kaimal AJ, Norton ME, Kuppermann M. Prenatal testing in the genomic age: Clinical outcomes, quality of life, and costs. Obstetrics and Gynecology. 2015; 126: 737-46.

36. Hulstaert F, Neyt M, Gyselaers W. The non-invasive prenatal test (NIPT) for trisomy 21—health economic aspects. Health Technology Assessment (HTA) Brussels: Belgian Health Care Knowledge Centre (KCE). 2014; 22: 2014.

37. Kostenko E, Chantraine F, Vandeweyer K, et al. Clinical and Economic Impact of Adopting Noninvasive Prenatal Testing as a Primary Screening Method for Fetal Aneuploidies in the General Pregnancy Population. Fetal Diagnosis and Therapy. 2019; 45: 413-23.

38. Morris S, Karlsen S, Chung N, Hill M, Chitty LS. Model-based analysis of costs and outcomes of non-invasive prenatal testing for Down's syndrome using cell free fetal DNA in the UK National Health Service. PloS one. 2014; 9: e93559-e59.

39. Neyt M, Hulstaert F, Gyselaers W. Introducing the non-invasive prenatal test for trisomy 21 in Belgium: A cost-consequences analysis. BMJ Open. 2014; 4.

40. Nshimyumukiza L, Menon S, Hina H, Rousseau F, Reinharz D. Cell‐free DNA noninvasive prenatal screening for aneuploidy versus conventional screening: A systematic review of economic evaluations. Clinical genetics. 2018; 94: 3-21.

41. Ökem ZG, Örgül G, Kasnakoglu BT, Çakar M, Beksaç MS. Economic analysis of prenatal screening strategies for Down syndrome in singleton pregnancies in Turkey. European Journal of Obstetrics and Gynecology and Reproductive Biology. 2017; 219: 40-44.

42. Okun N, Teitelbaum M, Huang T, Dewa CS, Hoch JS. The price of performance: a cost and performance analysis of the implementation of cell-free fetal DNA testing for Down syndrome in Ontario, Canada. Prenatal diagnosis. 2014; 34: 350-56.

43. O'Leary P, Maxwell S, Murch A, Hendrie D. Prenatal screening for Down syndrome in Australia: Costs and benefits of current and novel screening strategies. Australian and New Zealand Journal of Obstetrics and Gynaecology. 2013; 53: 425-33.

44. Shang W, Wan Y, Chen J, Du Y, Huang J. Introducing the non-invasive prenatal testing for detection of down syndrome in China: A cost-effectiveness analysis. BMJ Open. 2021; 11: e046582.

45. Song K, Musci TJ, Caughey AB. Clinical utility and cost of non-invasive prenatal testing with cfDNA analysis in high-risk women based on a US population. Journal of Maternal-Fetal and Neonatal Medicine. 2013; 26: 1180-85.

46. Walker BS, Nelson RE, Jackson BR, et al. A cost-effectiveness analysis of first trimester non-invasive prenatal screening for fetal trisomies in the United States. PLoS ONE. 2015; 10.

47. Wanapirak C, Buddhawongsa P, Himakalasa W, Sarnwong A, Tongsong T. Fetal Down syndrome screening models for developing countries; Part II: Cost-benefit analysis. BMC health services research. 2019; 19: 898.

48. Xie X, Wang M, Goh ESY, et al. Noninvasive Prenatal Testing for Trisomies 21, 18, and 13, Sex Chromosome Aneuploidies, and Microdeletions in Average-Risk Pregnancies: A Cost-Effectiveness Analysis. Journal of Obstetrics and Gynaecology Canada. 2020; 42: 740-49.e12.

49. Zhang W, Mohammadi T, Sou J, Anis AH. Cost-effectiveness of prenatal screening and diagnostic strategies for down syndrome: A microsimulation modeling analysis. PloS one. 2019; 14: e0225281-e81.

50. Zhang W, Mohammadi T, Sou J, Anis AH. Cost-effectiveness of prenatal screening and diagnostic strategies for down syndrome: A microsimulation modeling analysis. PLoS ONE. 2019; 14.

51. Harris RA, Washington AE, Nease RF, Kuppermann M. Cost utility of prenatal diagnosis and the risk-based threshold. The Lancet (British edition). 2004; 363: 276-82.

52. Mok WKY, Wong WH-S, Mok GTK, et al. Validation and application of health utilities index in Chinese subjects with down syndrome. Health and quality of life outcomes. 2014; 12: 144-44.

53. Maxwell S, Brameld K, Bower C, et al. Socio-demographic disparities in the uptake of prenatal screening and diagnosis in Western Australia. Australian & New Zealand journal of obstetrics & gynaecology. 2011; 51: 9-16.
